# Identification of imprinted genes subject to parent-of-origin specific expression in *Arabidopsis thaliana *seeds

**DOI:** 10.1186/1471-2229-11-113

**Published:** 2011-08-12

**Authors:** Peter C McKeown, Sylvia Laouielle-Duprat, Pjotr Prins, Philip Wolff, Marc W Schmid, Mark TA Donoghue, Antoine Fort, Dorota Duszynska, Aurélie Comte, Nga Thi Lao, Trevor J Wennblom, Geert Smant, Claudia Köhler, Ueli Grossniklaus, Charles Spillane

**Affiliations:** 1 Genetics and Biotechnology Lab, Botany and Plant Science, National University of Ireland Galway (NUIG), C306 Aras de Brun, University Road, Galway, Ireland; 2 Laboratory of Nematology, Wageningen University, Droevendaalsesteeg 1, Wageningen, The Netherlands; 3 Department of Biology and Zürich-Basel Plant Science Center, Swiss Federal Institute of Technology, ETH Centre, CH-8092 Zürich, Switzerland; 4 Department of Plant Biology and Forest Genetics, Uppsala BioCenter, Swedish University of Agricultural Sciences, SE-75007 Uppsala, Sweden; 5 Institute of Plant Biology and Zürich-Basel Plant Science Center, University of Zürich, Zollikerstrasse 107, CH-8008 Zürich, Switzerland; 6 Silicon Life Sciences, Minneapolis, MN, USA

## Abstract

**Background:**

Epigenetic regulation of gene dosage by genomic imprinting of some autosomal genes facilitates normal reproductive development in both mammals and flowering plants. While many imprinted genes have been identified and intensively studied in mammals, smaller numbers have been characterized in flowering plants, mostly in *Arabidopsis thaliana*. Identification of additional imprinted loci in flowering plants by genome-wide screening for parent-of-origin specific uniparental expression in seed tissues will facilitate our understanding of the origins and functions of imprinted genes in flowering plants.

**Results:**

cDNA-AFLP can detect allele-specific expression that is parent-of-origin dependent for expressed genes in which restriction site polymorphisms exist in the transcripts derived from each allele. Using a genome-wide cDNA-AFLP screen surveying allele-specific expression of 4500 transcript-derived fragments, we report the identification of 52 maternally expressed genes (MEGs) displaying parent-of-origin dependent expression patterns in Arabidopsis siliques containing F1 hybrid seeds (3, 4 and 5 days after pollination). We identified these MEGs by developing a bioinformatics tool (GenFrag) which can directly determine the identities of transcript-derived fragments from (i) their size and (ii) which selective nucleotides were added to the primers used to generate them. Hence, GenFrag facilitates increased throughput for genome-wide cDNA-AFLP fragment analyses. The 52 MEGs we identified were further filtered for high expression levels in the endosperm relative to the seed coat to identify the candidate genes most likely representing novel imprinted genes expressed in the endosperm of *Arabidopsis thaliana*. Expression in seed tissues of the three top-ranked candidate genes, *ATCDC48*, *PDE120 *and *MS5-like*, was confirmed by Laser-Capture Microdissection and qRT-PCR analysis. Maternal-specific expression of these genes in *Arabidopsis thaliana *F1 seeds was confirmed via allele-specific transcript analysis across a range of different accessions. Differentially methylated regions were identified adjacent to *ATCDC48 *and *PDE120*, which may represent candidate imprinting control regions. Finally, we demonstrate that expression levels of these three genes in vegetative tissues are *MET1*-dependent, while their uniparental maternal expression in the seed is not dependent on *MET1*.

**Conclusions:**

Using a cDNA-AFLP transcriptome profiling approach, we have identified three genes, *ATCDC48*, *PDE120 *and *MS5-like *which represent novel maternally expressed imprinted genes in the *Arabidopsis thaliana *seed. The extent of overlap between our cDNA-AFLP screen for maternally expressed imprinted genes, and other screens for imprinted and endosperm-expressed genes is discussed.

## Background

Flowering plant (angiosperm) seeds are chimeric structures which contain tissues whose cells have unequal genomic contributions from the maternal and paternal parents [1-3]. Within *Arabidopsis thaliana *seeds the diploid embryo is comprised of cells containing nuclear genomes inherited equally from the maternal and paternal parents. In contrast, the triploid endosperm contains two maternally inherited nuclear genomes and one paternal genome. In addition, these two fertilisation products are surrounded by a maternally derived diploid seed coat [4]. The triploid endosperm is a terminally differentiated structure which nourishes the developing embryo, while the diploid maternal seed coat plays key roles in supporting the development of the seed and the embryo it harbours [5]. The interactions between these different tissues and genomes during seed development in plants remain poorly understood [6,7], despite the fundamental economic importance of angiosperm seeds. For any given gene, the relative and absolute contribution of each seed tissue to overall transcript levels in the seed can be difficult to determine.

An important consequence of the unequal contributions of male and female genomes to the chimeric seed is that seed development can be affected by genome dosage and parent-of-origin effects [6,8,9]. Such maternal effects include sporophytic maternal effects from the maternally derived seed coat and gametophytic maternal effects derived from the female gametes. Gametophytic maternal effects on seed development can be due (a) to general dosage effects in the endosperm; (b) to deposition of maternal transcripts expressed prior to fertilization in the egg and central cell that give rise to the embryo and endosperm, respectively; or (c) to epigenetic regulation of genes via genomic imprinting, whereby autosomal genes are uniparentally expressed post-fertilisation in a parent-of-origin-specific manner [9,10].

Genomic imprinting has been predominantly described in mammals and flowering plants where it occurs in nutritive tissues (endosperm, placenta) and the developing embryo, although the latter is rare in plants [11]. While there are many theories regarding the evolution of genomic imprinting in mammals and plants, some focus on imprinting arising due to a 'parental conflict' over resource allocation [12,13] or due to a necessity to limit gene dosage of key genes during early development [14,15].

Many imprinted genes (i.e. hundreds, typically arranged in gene clusters along chromosomes) have been identified and intensively studied in mammalian species [16]. Until recently (2010), only 18 imprinted genes had been reported across all flowering plant species, 11 of them in *Arabidopsis thaliana *(Additional file 1 Table S1). Imprinted genes have been identified using a range of different strategies, including: mutant screens for maternally-controlled seed abortion (*Arabidopsis thaliana MEA *and *FIS2 *[17]); screens for genes regulated by the FIS *Polycomb *group complex (*Arabidopsis thaliana PHE1 *[18]); microarray analyses searching for genes showing similar responses to known imprinted genes (*Arabidopsis thaliana MPC *[19]); endosperm mRNA profiling (maize *nrp1 *[20]), and via a combination of microarray profiling and allele-specific expression analysis on endosperm from reciprocally crossed inbred lines (eight maize genes [21]). Using *cdka;1 *fertilized seeds which lack a paternal genome contribution to the (unfertilised) central cell, Shirzadi et al (2011) used microarray profiling to identify *AGL36 *as a maternally expressed imprinted gene amongst the 600 genes differentially regulated in the absence of a paternal genome [22]. The advent of next generation sequencing based transcriptomics has facilitated the recent identification of additional imprinted gene candidates in *Arabidopsis thaliana *seeds [23,24]. Hsieh et al (2011) [24] identified 43 confirmed imprinted genes (9 paternally expressed, 34 maternally expressed) in F1 hybrid seeds (7-8 days after pollination) from L*er*-0 × Col-0 reciprocal crosses. Again using next generation sequencing approaches, Wolff et al (2011) [23] have identified 65 candidate imprinted genes in F1 hybrid seeds (4 days after pollination) from Bur-0 × Col-0 reciprocal crosses of which 19 were confirmed in both cross directions (8 paternally expressed, and 11 maternally expressed). Hence, 'next generation' sequencing studies are now being employed to identify putative imprinted genes [23,24].

An indirect approach for the identification of novel imprinted genes has been conducted based on identification of differentially methylated regions (DMRs) as candidate imprinting control regions (ICRs) [25]. Genes acting as modifiers of genomic imprinting have also been identified in plants and include *MET1 *[26], *DDM1 *[17] and *DME *[27]. For example, the 5-methylcytosine DNA glycosylase gene *DME *is preferentially expressed in the central cell of the female gametophyte and can regulate the expression of some imprinted genes in the endosperm through demethylation of their ICRs [27]. In mutant *dme *endosperm ICRs remain methylated and as a result some imprinted genes are misregulated, which facilitates their detection [27].

While there are a number of genome-wide profiling approaches that can be used to identify allele-specific expression, there are several significant challenges for the definition of novel imprinted genes [28]. To distinguish between allele-specific expression effects that are either parent-of-origin dependent (e.g. imprinting) or independent, it is necessary to demonstrate the parent-of-origin dependency of uniparental expression at imprinted loci by analysis of reciprocal F1 hybrid offspring. Furthermore, where maternal-specific expression is detected in a plant seed, it is necessary to distinguish between seed coat *versus *endosperm (and/or embryo) expression, and also to distinguish between transcripts maternally deposited in the egg and/or central cell *versus *transcripts generated post-fertilisation in the developing endosperm and/or embryo [11]. While imprinted genes displaying clear mutant phenotypes (e.g. *medea*) on seed development can facilitate interpretation of such loci as imprinted [10], many of the imprinted genes identified to date do not display any obvious mutant phenotype in seeds [29]. In some instances, promoter:reporter constructs have been used to identify *cis*-regulatory regions that are required for imprinting [19,30], while only one study has demonstrated post-fertilisation nascent uniparental *de novo *transcription of an imprinted gene in the endosperm [17].

The choice of transcript profiling platform is an important consideration for identification of novel imprinted genes. Microarrays are dependent on genes being expressed at a level sufficient to be detectable via hybridization and complementary strategies are necessary to also detect imprinted genes that may be lowly expressed. Hence, in this study we chose cDNA-AFLP [31] for genome-wide screening for novel imprinted genes. Although an early generation transcript profiling technology, as a PCR-based technology, cDNA-AFLP allows the amplification of even lowly expressed transcripts and can identify uniparentally expressed transcripts for all cases where there is a restriction site polymorphism between the parental alleles. To facilitate genome-wide cDNA-AFLP expression profiling, we have developed a gene-identifying bioinformatic software program, GenFrag, which can determine the identity of genes displaying parent-of-origin specific cDNA-AFLP expression profiles.

Our analysis of allele-specific expression of 4500 transcript-derived fragments (TDFs) in an experimental design based on the generation of reciprocal F1 hybrids seeds allowed the identification of 52 genes displaying maternal-specific expression (MEGs). The maternal specific expression of some of these MEGs may be due to genomic imprinting. Within these 52 maternally expressed genes, 18 represent genes that display higher relative and absolute expression levels in the endosperm relative to the maternal seed coat. Hence, the detection of maternal-specific expression of such genes in F1 hybrid seeds 4 days after pollination (dap) is consistent with such genes being subject to genomic imprinting in the developing endosperm. Four of these 18 MEGs have proximal differentially methylated regions (DMRs) in seed endosperm from wild-type and *dme *mutant backgrounds that may represent candidate imprinting control elements (ICRs). For the three top ranked candidates (*ATCDC48, PDE120 *and *MS5-like) *we confirm maternal-specific expression in F1 hybrid seeds 4 dap and characterise the control of their allele-specific expression at different developmental stages, and in different genetic and mutant backgrounds. Overall, we have identified a range of novel MEGs in *Arabidopsis thaliana *seeds, from which we further demonstrate that three are novel maternally expressed imprinted genes in *Arabidopsis thaliana *seeds.

## Results

### cDNA-AFLP expression profiling of *Arabidopsis thaliana *siliques containing F1 hybrid seeds detects 93 uniparentally-expressed TDFs

To identify genes which are uniparentally expressed in F1 hybrid seeds within siliques of *Arabidopsis thaliana *we employed a genome-wide cDNA-AFLP transcriptome profiling approach. At 3, 4 and 5 dap, RNA samples were generated from siliques containing F1 hybrid seeds generated via reciprocal crosses between the accessions Col-0 and Ler-0. These three stages correspond to developmental stages from the late globular (3 dap) to early and late heart stages (4 and 5 dap) of embryo development within the seed. These stages of embryo development were chosen to mitigate against the possibility of detection of maternally deposited long-lived RNAs in the egg cell and/or central cell, and also because zygotic expression from both parental alleles is evident at these developmental stages [32]. In these samples, maternally expressed genes may be detected from either the silique or F1 seed tissues, and within the F1 seeds from either the maternal seed coat or the fertilisation products (i.e. the embryo and/or endosperm).

AFLP was performed on cDNA derived from RNA samples following restriction digestion with a frequently cutting enzyme (*Bst*YI) and a rare cutting enzyme (*Mse*I) (Additional file 2 Figure S1). Fragments were ligated with adapters complementary to the restriction sites of the enzymes. To reduce the complexity of the mixture of fragments, a series of PCR amplifications were performed to generate subsets of fragments using selective primers. These selective primers share a common sequence, which corresponds to the adapters and a section of the restriction sites but are differentiated by one or two additional nucleotides at the 3'end, called selective nucleotides (Methods; Additional file 2 Figure S1).

The cDNA-AFLP generated transcript derived fragments (TDFs) were run on an ABI3130xl capillary analyser and visualized with fluorescently labelled probes to accurately estimate their size (see Methods). A total of 10,200 TDFs were detected across the three time points (3, 4, 5 dap). The TDFs ranged in size from 50 to 500 base pairs (bp) and an average of 80 bp was visualized per sample. Of the 10,200 TDFs screened, 4500 showed a polymorphism between cDNA derived from the reciprocal crosses between the two different accessions (genetic backgrounds) with sizes ranging from 100 bp to 500 bp. Maternally expressed alleles were found in approximately equal numbers when each of the two accessions were used as the maternal parent in a reciprocal cross (Additional file 3 Table S2). For example, at the 4 dap time-point, 366 maternally expressed Col-0 alleles were detected in the Col-0 × Ler-0 cross, while 306 maternally expressed Ler-0 alleles were detected in the reciprocal Ler-0 × Col-0 cross. The numbers of maternally expressed TDFs detected were similar across the three developmental stages indicating consistency of maternal-specific transcription during early silique development. For each polymorphic allele (i.e. Col-0 vs Ler-0 alleles differing in a restriction site), only one fragment is detectable from each restriction digestion event as only those TDFs proximal to the poly-A tail were isolated for analysis. Hence for each of the two accessions there is no redundancy within the number of TDFs detected at each time-point.

To identify uniparentally expressed genes, cDNA-AFLP profiles for these 4500 polymorphic TDFs were compared between those obtained from siliques containing reciprocal F1 hybrid seeds (i.e. F1 progeny of Ler-0 × Col-0 versus Col-0 × Ler-0 crosses) and those obtained from the equivalent cross between plants of the same accession (i.e. Col-0 × Col-0, Ler-0 × Ler-0). The samples at 3, 4 and 5 dap were used to filter for TDFs which displayed uniparental expression for at least two of the stages sampled. This strategy allowed the identification of 93 uniparentally expressed TDFs. All 93 of the uniparentally expressed TDFs displayed a maternal-specific expression pattern (Additional file 4 Table S3).

### Direct identification of genes based on TDF size and the selective nucleotides of each primer combination using the GenFrag bioinformatics program

To identify the genes that produced the maternal TDFs detected in *Arabidopsis thaliana *siliques containing F1 hybrid seeds (Additional file 4 Table S3), we developed a bioinformatics program called GenFrag. GenFrag is designed to allow *in silico *identification of sequences of TDFs produced by cDNA-AFLP using publicly available cDNA and EST libraries (which for the well annotated *Arabidopsis thaliana *genome also includes all curated alternative splice variants [33]). Using these resources, GenFrag is designed to simulate the steps of the cDNA-AFLP *in silico *by scanning existing *Arabidopsis thaliana *genome information for dual restriction enzyme cutting sites (see Methods and Additional file 2 Figure S1). Given the fragment size (as assessed on the capillary sequencer) and the selective nucleotides added to the primers used to generate the TDF, GenFrag can identify the corresponding sequence of a TDF and thereby the identity of the gene corresponding to the TDF. The GenFrag software is developed as open source software and is freely available for use online at: http://www.nem.wur.nl/UK/Research/bio/.

### GenFrag-based identification of 52 genes from the set of 93 maternally expressed TDFs

GenFrag was used to identify genes corresponding to the 93 maternal specific TDFs (Additional file 4**Table S3**). To increase selectivity, we incorporated an option into GenFrag to only return the last matched fragment in a 5'-3' sequence i.e. the fragment closest to the poly-A tail of the mRNA. We combined this adaptation with a stringent range of 1 bp deviation between the observed size of the TDF when run on the capillary analyser and the size predicted *in silico *for a candidate sequence. Using these conditions, GenFrag was able to determine unique sequence (i.e. gene ID) matches for 52 of the 93 maternally expressed TDFs identified (i.e. TDFs 1-52 in Additional file 4 Table S3). Of the remaining TDFs, 21 matched sequences shared by more than one gene and therefore could not be uniquely distinguished (TDFs 53-73 in Additional file 4 Table S3), while the remaining 20 could not be matched to any genes using the GenFrag approach (TDFs 74-93 in Additional file 4 Table S3). The lack of identification of these 20 TDFs may be due to aberrant enzyme restriction and/or incomplete coverage of the *Arabidopsis thaliana *transcriptome. The 52 unique sequence TDFs were matched to genes by BLAST searching the *Arabidopsis thaliana *genome (TAIR v.8). This allowed us to unambiguously identify 52 maternally expressed genes in *Arabidopsis thaliana *siliques containing F1 hybrid seeds (Table 1). Gene Ontology enrichment analysis of the 52 maternally expressed genes did not reveal any significant enriched terms (data not shown). Our set of 52 MEGs did not include the known imprinted genes from *Arabidopsis thaliana*, however, this is not surprising as most of these 52 MEGs have few SNP differences between the alleles from different accessions, and where they do, the SNPs do not disrupt the restriction sites that are scanned by the cDNA-AFLP technique using these restriction enzymes (Additional file 5 Table S4). For instance, there are no Col-0/Ler-0 SNPs in the coding sequence of the maternally expressed imprinted gene *MEDEA*. The 52 genes we identify represent novel maternally expressed genes (MEGs).

**Table 1:** 52 genes are identified as maternally expressed by GenFrag analysis of cDNA-AFLP TDFs sizes and the selective nucleotides of the primer combinations used to generate the TDFs.

Gene	Protein encoded
At1g03070	Glutamate binding protein

At1g04700	Protein kinase family protein

At1g09390	GDSL-motif lipase/hydrolase family protein

At1g12420	ACT Domain Repeat 8 (ACR8)

At1g14880	Unknown protein

At1g16730	Unknown protein

At1g17840	ABC transporter family protein

At1g31820	Amino acid permease family protein

At1g54710	AtATG18

At1g55320	Ligase, similar to acyl-activating enzyme 17 (AAE17)

At1g61990	Mitochondrial transcription termination factor-related

At1g65820	Microsomal glutathione s-transferase, putative

At1g73680	Pathogen-responsive alpha-dioxygenase

At1g74450	Unknown protein

At1g75680	*Arabidopsis thaliana *glycosyl hydrolase 9B7 (ATGH9B7)

**At2g16480**	Unknown protein

**At2g21130**	Cyclophilin-like

At2g26620	Glycoside hydrolase family 28 protein

At2g31510	ARIADNE-like protein ARI7 (ARI7)

At2g32000	DNA topoisomerase family protein

At2g36020	Abscisic acid-responsive HVA22 family protein

At2g40810	*Arabidopsis thaliana *homolog of yeast autophagy 18c (ATG18c)

At2g45315	Unknown

At3g09840	Cell division cycle 48 (ATCDC48)

**At3g12370**	Mitochondrial RPL10

At3g20760	Nse4, component of Smc5/6 DNA repair complex

At3g22260	Ovarian tumor domain-like cysteine protease family protein

At3g24780	Uncharacterised conserved protein

At3g25530	Gamma-hydroxybutyrate dehydrogenase (ATGHBDH)

At3g29360	UDP-glucose 6-dehydrogenase

At3g47250	Unknown protein

**At3g51280**	Similar to male sterility MS5

At3g55250	Similar to calcium homeostasis regulator (CHoR1)

At3g57510	Arabidopsis endo-polygalacturonase 1 (ADPG1)

At3g59380	FARNESYLTRANSFERASE A (FTA)

At4g00180	YABBY gene family member

At4g01000	Ubiquitin family protein

At4g16830	Nuclear RNA-binding protein (RGGA)

**At4g21270**	AT KINESIN 1

At4g29450	Leucine-rich repeat protein kinase, putative

At4g33450	Myb domain protein 69 (AtMYB69)

At4g37530	Peroxidase, putative

**At5g04895**	ATP binding/helicase/nucleic acid binding protein

**At5g16620**	Pigment defective embryo (PDE120) chloroplast import (Tic40)

At5g17080	Cathepsin-related protein

At5g35730	EXS family protein/ERD1/XPR1/SYG1 family protein

At5g35737	Unknown protein

At5g38320	Unknown protein

At5g39510	VESICLE TRANSPORT V-SNARE 11 (VTI11)

At5g40390	Seed imbibition 1 (SIP1)

**At5g61300**	Unknown protein

At5g56310	ATHB5

52 maternally-expressed genes were identified from transcript-derived fragments generated by cDNA-AFLP of hybrid *A. thaliana *siliques. 93 TDFs were identified using GenFrag on the basis of their size and the selective nucleotides of the primer combinations used to generate them. These were matched to the 52 genes listed by BLASTN against *A. thaliana *genome (TAIR v.8). Nine genes which have been reported as preferentially endosperm-enriched (Day et al., 2008) are marked in bold.

### 18 candidate imprinted genes in which the observed maternal expression is predominantly derived from higher transcript levels in the endosperm relative to the maternal seed coat

The 52 maternally expressed genes (MEGs) were detected in siliques containing reciprocal F1 hybrid seeds where the maternal-specific expression could be derived from the silique, the maternal seed coat, the endosperm and/or the embryo. Seed expressed genes which are predominantly maternally expressed in the endosperm from 3 dap (late globular stage embryos) are excellent candidates for regulation by genomic imprinting. It was recently shown that embryo development up to the globular stage does not depend on *de novo *transcription while endosperm development requires active transcription following fertilization, suggesting that maternally deposited RNAs do not play a predominant role in the endosperm [34]. Thus, mRNAs detected in the endosperm at ≥ 3 dap are most likely to be derived from *de novo *transcription post-fertilization. To identify which of the 52 maternally expressed genes are predominantly expressed in the endosperm at high expression levels, we used a publicly available expression dataset (Seed Gene Network - Harada-Goldberg Arabidopsis Laser Capture Microdissection Gene Chip Data Set, http://seedgenenetwork.net; [35]) where the relative expression levels of genes in the seed coat and endosperm tissues (peripheral, chalazal and micropylar fractions) of seeds at the globular stage of embryo development (3 dap) have been assessed.

From the 52 maternally expressed genes, we could identify 32 genes which had strong signals of expression in the 3 dap seed. Eleven genes were not detected as they did not have probes in the array dataset used, or their probes also matched another gene. Nine genes were not expressed in seeds and therefore may be good candidates for silique specific MEGs. Comparing the expression levels between the endosperm and the seed coat, we found three MEGs which were exclusively expressed in the seed coat but no MEGs which were absent from the seed coat but were expressed in the endosperm. However, twenty-nine MEGs showed expression in both the endosperm and the seed coat. We considered that if maternal-specific expression can be demonstrated in seeds for MEGs where the majority of the expression level signal is from the endosperm, that such a pattern would be strongly indicative of a maternally expressed imprinted gene in the endosperm. Biallelic expression in the endosperm should also be easier to detect in such cases. Hence, for these twenty-nine MEGs, we aimed to identify genes where the majority of the expression detected in the seed is due to the endosperm fraction. We selected the 18 genes out of the 29 that showed higher expression in the endosperm compared to the seed coat and ranked these genes based on the absolute difference of expression levels between the highest expressing endosperm fraction and the seed coat (Table 2). We reasoned that genes displaying the highest levels of expression in the endosperm of 3 dap seeds were least likely to be genes where maternal-specific transcripts detected could be due to maternal deposition of transcripts in the central cell [34] or transferred from the maternal seed coat as has recently been proposed [24] i.e. we focussed on genes which are highly expressed in the endosperm relative to the maternal seed coat. As a complementary approach, we also compared these genes on the basis of relative transcription levels (Additional file 6 Table S5). For these MEGs with significantly higher expression levels in the endosperm when compared to the seed coat, maternal-specific expression detected in reciprocal F1 hybrid seeds at 4 dap is consistent with regulation via genomic imprinting in the endosperm. Using these approaches, we chose the three top ranked genes as measured by total enrichment of expression in the endosperm, *ATCDC48 *(At3g09840), *PDE120 *(At5g16620) and *MS5-like *(At3g51280) as our strongest imprinted candidates for further investigation. Although *PDE120 *and *MS5-like *were less highly expressed in the endosperm in total, they were also the most highly ranked genes as measured by ratio of endosperm:seed coat expression (Additional file 6 Table S5) and as noted in Table 1 have previously been reported as preferentially endosperm-expressed in a microarray study performed by Day et al. [36]. Hence we consider all three of these MEGs to be principally expressed in the F1 endosperm relative to the maternal seed coat.

**Table 2:** Maternally expressed genes ranked by absolute expression level difference between highest-expressing endosperm fraction and seed coat

Gene ID	Seed coat expression level	Embryo expression level	Peripheral endosperm expression level	Micropylar endosperm expression level	Chalazal endosperm expression level	Absolute difference of expression levels between highest-expressing endosperm fraction and seed coat (hEF-SC)	Ratio of expression levels between highest-expressing endosperm fraction and seed coat (hEF/SC)
At3g09840 (*AtCDC48A*)	9462.69	12859.55	8565.74	7199.95	15983.67 *	6520.97	1.69

At5g16620 (*PDE120*)	1882.19	3721.39	7328.89 *	1547.72	594.65	5446.69	3.89

At3g51280 (*MS5*)	143.71	6909.54	3598.61 *	425.12	170.39	3454.9	25.04

At4g16830	1403.41	2234.66	3777.26 *	3520.61	1358.85	2373.85	2.69

At5g63330	364.44	215.12	512.38	340.48	1942.53 *	1578.09	5.33

At1g73680	2286.18	68.31	1281.93	3787.4 *	1095.28	1501.21	1.66

At1g03070	150.95	22.77	43.96	70.68	1273.49 *	1122.55	8.44

At3g24530	839.73	1359.11	1940.84 *	1502.07	352.68	1101.11	2.31

At1g65820	757.13	253.23	413.46	1813.32 *	612.73	1056.2	2.39

At3g17000	416.34	137.53	165.76	440.71	1401.76 *	985.42	3.37

At5g39510	1934.73	812.5	1357.99	2472.39 *	2071.39	537.65	1.28

At1g25370	362.23	56.59	52.88	339.09	718.4 *	356.17	1.98

At3g59380	333.7	299	481.63	597.3	631.7 *	298.01	1.89

At3g55250	183.62	264.14	461.66 *	219.78	83.6	278.04	2.51

At2g31510	398.3	455.28	416.09	642.15 *	195.12	243.85	1.61

At2g16480	620.32	793.04	854.07 *	611.2	577.62	233.75	1.38

At1g61990	265.14	479.11	335.01	403.18	470.95 *	205.81	1.78

At2g32000	280.44	244.55	333.14 *	176.92	98.83	52.7	1.19

Expression levels in *Arabidopsis thaliana *seed coat (SC), embryo and peripheral, micropylar and chalazal endosperm tissues of 18 maternally expressed genes. * highlights the highest-expressing endosperm fraction (hEF). Microarray data is from Seedgenenetwork (Harada-Goldberg Arabidopsis Laser Capture Microdissection Gene Chip Data Set, http://seedgenenetwork.net).

### Laser capture microdissection (LCM) and qRT-PCR confirm expression of *ATCDC48, PDE120 *and *MS5-like *in *Arabidopsis thaliana *seed

To validate the expression patterns of the three top ranked imprinted gene candidates *ATCDC48*, *PDE120 *and *MS5-like*, we used Laser Capture Microdissection (LCM) to microdissect *Arabidopsis thaliana *seeds (5 dap) of accession L*er*-0 into endosperm (ES), seed coat (SC) and embryo (EM) fractions. The three LCM tissues were screened by qualitative end-point RT-PCR to investigate tissue-specific expression of each gene within the seed at 5 dap, which confirmed that all three genes are indeed expressed in *Arabidopsis thaliana *seeds (Additional file 7 Figure S2). Transcripts were detected in both the seed coat and endosperm for all three genes, while *ATCDC48 *and *MS5-like *were also detected in the embryo. Although this qualitative RT-PCR analysis provided no indication of relative expression levels in each of the three distinct parts of the seed, it served to independently confirm that the three genes are indeed expressed in seed tissues at 5 dap in the tissues predicted by the Seed Gene Network expression database (Table 2).

**Figure 1:**
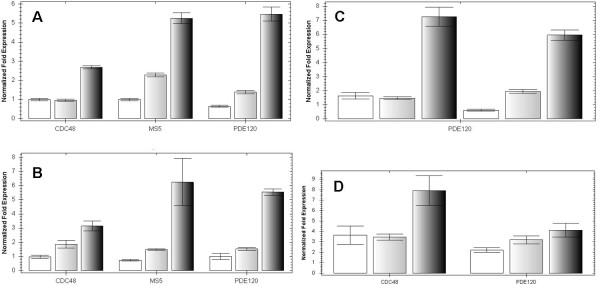
**Expression profiles of candidate imprinted genes in ***Arabidopsis thaliana ***seed as determined by qRT-PCR**. **1A**. Expression of *AtCDC48A, MS5-like *and *PDE120 *increases though Col-0 seed development at 3 dap (left-hand columns), 4 dap (middle columns) and 5-6 dap (right-hand columns) **1B**. Expression of *AtCDC48A, MS5-like *and *PDE120 *increases though Ler-0 seed development at 3 dap (left-hand columns), 4 dap (middle columns) and 5-6 dap (right-hand columns) **1C**. *PDE120 *is expressed in hybrid seeds in similar patterns to non-hybrid seeds. Determined at 3, 4 and 5-6 dap for Col-0 × L*er*-0 (first 3 columns) and L*er*-0 × Col-0 (second three columns). **1D**. *AtCDC48A *and *PDE120 *are expressed only at low levels in ovules of Col-0 (left-hand columns) or L*er*-0 (middle columns) compared to Col-0 4 dap seed (right-hand columns). Standard errors are shown.

To determine how the expression levels of these genes in seeds varied over the time-course covered by our cDNA-AFLP experiment, we performed qRT-PCR on seeds at different time-points 3, 4 and 5-6 days after manual pollination. The existing data for whole-seed expression levels in Ws-0 (Seed Gene Network, [35]) predicted that expression of *MS5-like *and *CDC48A *would increase through development (across globular, heart and elongated cotyledon stages). In our qRT-PCR analysis, we found that this expression pattern was conserved in both Col-0 and L*er*-0 seeds (Figure 1A, B) indicating that for these genes there is little effect of accession background on total expression levels. However, we also found increased expression of *PDE120 *at the 5-6 dap time-point in both accessions, which differed from the Ws-0 data (Seed Gene Network) (Figure 1A, B).

To preclude any differences on expression levels that could be due to a hybrid background, we also measured expression of *PDE120 *within reciprocal Col-0 × L*er*-0 crosses at the 3, 4 and 5-6 dap time-points and again found increased expression through seed development (Figure 1C). This suggests that the expression patterns of these three seed-expressed genes, which are similar in both parental accessions, are not significantly altered in their F1 hybrid offspring, although transcript levels of *PDE120 *might be slightly higher at 3 dap in the Col-0 × L*er*-0 cross direction. Because expression increases throughout development, and was, in contrast, lower in pre-fertilized ovules (Figure 1D), this suggests that the expression we have detected is due to *de novo *post-fertilisation transcription and not maternal deposition of long-lived RNA transcripts from the central cell and/or egg cell to the post-fertilisation endosperm and/or embryo, respectively.

### The maternally expressed seed genes *ATCDC48*, *PDE120 *and *MS5-like *are subject to gene-specific imprinting in different genetic backgrounds

**Figure 2:**
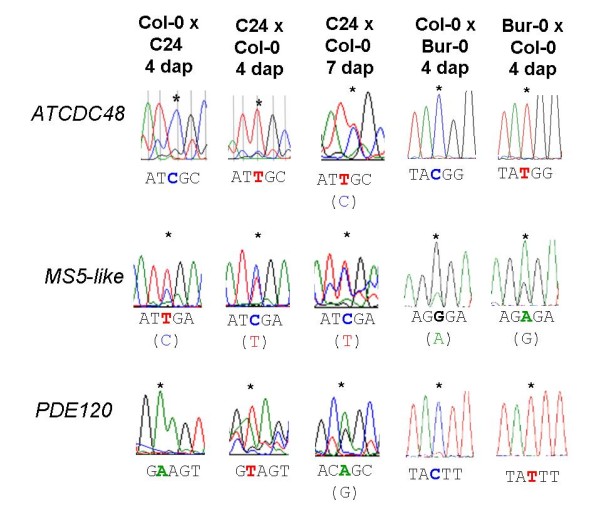
***ATCDC48*, *PDE120 *and *MS5-like *are expressed from the maternal allele in *Arabidopsis thaliana *F1 hybrid seeds (4 dap)**. Allele-specific sequencing of *ATCDC48*, *PDE120 *and *MS5-like *from crosses between different Arabidopsis F1 seeds formed by hybridizing different accessions at 4 dap when only the maternal alleles are represented in the sequences directions; and of Col-0 × C24 F1 seeds at 7 dap, when the paternal allele is becoming expressed. Positions of SNPs are marked by asterisks and the relevant maternal allele listed below each trace.

Genomic imprinting can be 'gene-specific' (where all alleles of the gene are imprinted in the majority of genetic backgrounds) or 'allele-specific' (where only one or a few alleles are imprinted in specific genetic backgrounds) [28]. To validate the three top-ranked genes as maternally expressed imprinted genes and to test for gene- vs allele-specific imprinting, we identified SNPs in the coding regions of each gene between the Col-0 and C24 accessions, and between the Col-0 and Bur-0 accessions. We sequenced cDNA from reciprocal F1 hybrid seeds (4 dap) to detect any evidence of mono-allelic expression patterns consistent with regulation of the genes by genomic imprinting. To confirm the effects in both of the genetic backgrounds used for cDNA-AFLP, we also sequenced SNPs in cDNA from F1 hybrid seeds (4 dap) of L*er*-0 × Col-0 crosses for *PDE120 *and *MS5-like*. In all cases, we found that *ATCDC48*, *PDE120 *and *MS5-lik*e were maternally expressed in F1 hybrid seeds at 4 dap (Figure 2; Additional file 8 Figure S3). While binary imprinted expression (on/off) was observed for *ATCDC48 *and *PDE120*, *MS5-like *displayed preferential expression of the maternally inherited allele (Figure 2). This indicates that the imprinted status of these three genes, like their expression levels (Figure 1), is conserved across divergent accessions and that they likely represent cases of gene-specific imprinting.

As a more general validation of the cDNA-AFLP approach to detect maternally expressed seed genes, we chose six further genes predicted to be expressed in seed tissues and sequenced SNPs in cDNA generated from Col-0 × C24 and C24 × Col-0 F1 hybrid seeds at 4 dap. In all six cases, we validated maternal-specific expression. We have therefore validated 9/52 = 17% of the genes identified as uniparentally expressed by cDNA-AFLP as MEGs (Additional File 9 Figure S4).

**Figure 3:**
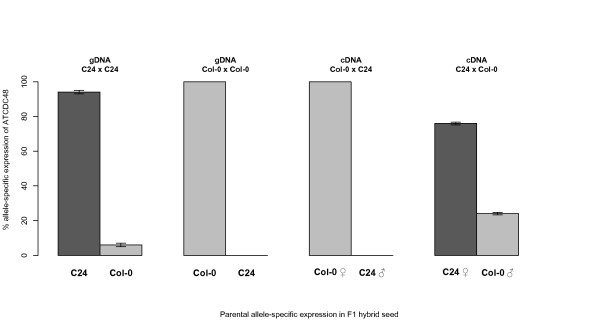
**Relative quantification of maternal and paternal transcripts for ***ATCDC48 ***in ***Arabidopsis thaliana ***F1 hybrid seeds (4 dap)**. Transcript expression levels of maternal and paternal alleles of *ATCDC48 *were quantified by QUASEP pyrosequencing of cDNA from reciprocal Col-0 × C24 F1 hybrid seeds at 4 dap. Genomic DNA from each parent was used as an assay control.

For the top ranked imprinted gene *ATCDC48*, we also quantified the extent of imprinting using Quantification of Allele Specific Expression by Pyrosequencing (QUASEP), a technique based on real-time pyrophosphate (PP_i_) detection [32-34], which allows precise relative quantification of SNP frequencies (Figure 3). QUASEP was performed on the maternally expressed imprinted gene *ATCDC4*8 using cDNA collected from reciprocal Col-0 × C24 F1 hybrid seeds (4 dap). The known imprinted genes *FWA *and *PHE1 *were used as controls (Table 3), which confirmed maternal-specific (binary) and paternal-specific (preferential) expression patterns for these two imprinted genes, respectively [26,37]. *PHE2*, the non-imprinted endosperm-expressed homologue of *PHE1*, was used as a biallelic control (Table 3). We found that in F1 hybrid seeds at 4 dap the relative expression level from the maternally inherited allele of *ATCDC48 *was 100% (Col-0 × C24) and 80.5% (C24 × Col-0) indicating that *ATCDC48 *displays maternal-specific expression (Figure 2). Although *ATCDC48 *is subject to expression in the seed coat, it displays high expression levels in the chalazal endosperm (Table 2), which is consistent with post-fertilisation transcription in the endosperm rather than a scenario of deposition of maternal transcripts in the central cell. Thus, the expression pattern of *ATCDC48 *is consistent with *ATCDC48 *being a novel maternally expressed imprinted gene in the endosperm of *Arabidopsis thaliana *seeds.

**Table 3:** Comparative controls for quantification of maternal expression of *ATCDC48A *by QUASEP.

Gene	Name	SNP	Col-0 allele (maternal)	C24/L*er*-0 allele (maternal	Mean maternal	Imprinted status
At3g09840	*CDC48A*	Col-0/C24	100.0%	80.5%	90.4%	MEG test gene

At4g25530	*FWA *	Col-0/Ler*-*0	92.1%	97.1%	94.6%	MEG control gene

At1g65330	*PHE1*	Col-0/Ler*-*0	27.4%	12.8%	21.1%	PEG control gene

At1g65330	*PHE2*	Col-0/Ler*-*0	56.3%	35.3%	45.8%	Biallelic control

*FWA *and *PHERES1 *were used as maternally and paternally expressed controls, respectively; the non-imprinted gene, *PHERES2 *was as a control expressed from both alleles within the endosperm.

Both *ATCDC48 *and *MS5-like *also show high levels of expression in the embryo (Table 2). Biallelic expression at the heart stage of embryo development would be expected for most embryo-expressed genes, following the earlier reactivation of the paternal genome (from the globular embryo stage onwards) in *Arabidopsis thaliana *[32]. In the case of *MS5-like*, expression within the seed is largely confined to the embryo and to the peripheral endosperm. It is likely that imprinting of *MS5-like *occurs exclusively within the 4 dap endosperm whilst expression in the embryo is biallelic, which could explain the partial peak of expression from the paternal allele of this gene (Figure 2). For *ATCDC48 *however, the detection of almost exclusively maternal transcripts by sequencing and QUASEP could suggest that *ATCDC48 *may undergo delayed reactivation of the paternally inherited allele in the 4 dap embryo.

### Expression of imprinted genes in endosperm of seeds at later developmental stages

In a recent study, Hsieh et al. (2011) [24] screened for novel imprinted genes in 7-8 dap seed from reciprocal crosses between Col-0 and L*er*-0. The differences between the numbers of uniparental TDFs identified by cDNA-AFLP at 3, 4 and 5 dap (Additional file 2 Table S2), with only 92 uniparental TDFs detected at multiple developmental stages, suggests some temporal dynamism in the regulation of imprinting in *Arabidopsis thaliana *seeds which could potentially explain the lack of overlap between our results and those of Hsieh et al. [24]. To test this, we investigated whether the MEGs we had identified at 4 dap remained monoallelic or became biallelic at later developmental stages. Our results indicate that in cDNA from 7 dap seed, paternal alleles were more highly expressed than at 4 dap for all three of the genes (Figure 2). In the case of *ATCDC48A*, this rendered the expression fully biallelic, whilst the maternal allele was still preferentially expressed for *MS5-like *and *PDE120 *(Figure 2). At the 7 dap time-point, while all three genes are expressed from the embryo and endosperm, the relative and absolute contributions of each tissue to total transcript levels in the 7 dap seed are not known. Hence, the increased expression of the paternal allele observed in the 7 dap seed could arise from loss of imprinting and/or a shift in the relative proportion of embryo versus endosperm tissues amounts in the 7 dap seed (compared to the 4 dap seed). In the latter scenario, the MEG could remain imprinted in the endosperm tissue, but be masked by a biallelic expression signal from the more abundant embryo tissue at 7 dap. The expression of both alleles would be likely to preclude their identification at the p<0.001 cut-off used for most gene identifications by Hsieh et al. [24]. We also considered the concordance between our dataset and a further next-generation sequencing screen performed by Wolff et al. [23] (Additional File 10 Figure S5) and found no overlap either with our screen or with that of Hsieh et al. [24] (see also Discussion). We also found very little overlap (seven out of 100) between imprinted genes detected by these two studies and differentially methylated regions (DMRs) previously predicted by Gehring et al. [25]. This prompted us to consider the possible existence of unidentified DMRs which could act as imprinting control regions (ICRs) associated with our imprinted genes.

### Identification of DMRs at the *ATCDC48*, *PDE120 *and *MS5-like *loci

While the imprinting control regions (ICRs) of imprinted genes in mammals often overlap with differentially methylated regions (DMRs), the genome-wide distribution of DMRs means that only some of these are likely to be ICRs [38-41]. In plant genomes, ICRs that coincide with DMRs have been identified for the imprinted genes *FWA *[26,42], *PHE1 *[30], and *MPC *[19]. As noted above, however, they have not been detected for many other imprinted genes, and induction of imprinting by many putative DMRs [11] remains unconfirmed (Additional File 10 Figure S5). Using available methylation data for wild-type and *dme *endosperm [43], we searched for DMRs in the genomic vicinity of the maternally expressed imprinted loci *ATCDC48*, *PDE120 *and *MS5-like*.

**Figure 4:**
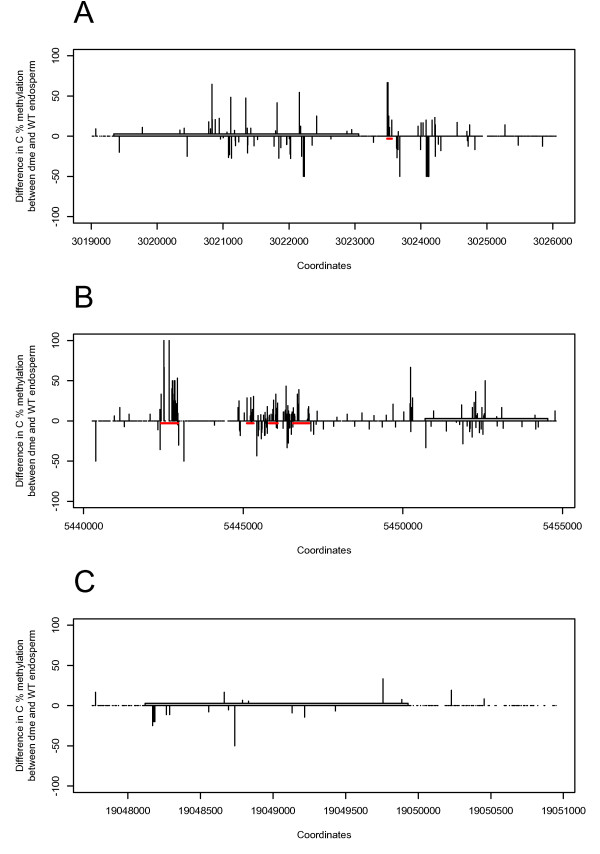
**DMRs are located in the vicinity of the *ATCDC48 *and *PDE120 *loci**. Difference in methylation percentage between *dme *and wild-type endosperm for cytosines in the vicinity of the *ATCDC48 *(**A**), *PDE120 *(**B**) and *MS5-like *(**C**) loci. Grey boxes represents the gene body (5'-3' orientation), red bars highlight DMRs with potential to act as ICRs.

We identified DMRs that could potentially act as ICRs for *PDE120 *and *ATCDC48 *(Figures 4A and 4B) by analysing expression data derived from endosperm of the wild type and endosperm of seeds deficient for a maternal *DME *allele [43]. These were retrieved from ArrayExpress and the percentage of methylation at cytosines situated between the genes immediately upstream and downstream of the gene bodies calculated. A DMR was located 432 bp downstream of *ATCDC48A *containing 26 cytosines, of which 6 are hypermethylated in *dme *(Figure 4A). Four DMRs were located upstream of *PDE120 *at distances of 8273 bp (30 cytosines, 17 hypermethylated in *dme*), 5377 bp (49 cytosines, 6 hypermethylated in *dme*), 4620 bp (46 cytosines, 13 hypermethylated in *dme*) and 3635 bp (115 cytosines, 12 hypermethylated in *dme*) (Figure 4B). No obvious *DME-*dependent DMRs could be identified in the genomic neighbourhood of the imprinted gene *MS5-like *(Figure 4C). We also analysed our entire portfolio of candidate imprinted genes (Table 2) for potential DMRs in their vicinity. In contrast to our three top ranked imprinted genes, we could only identify DMRs for two additional genes out of the other 49, namely At1g25370 (encoding a protein of unknown function containing a DUF1639) and At2g32000 (encoding a DNA topoisomerase, type 1A) (Additional File 11 Figure S6). Overall, these data suggest that the imprinted *MS5-like *gene is less likely to be regulated via a methylation-dependent mechanism than the imprinted genes *ATCDC48 *and *PDE120*.

### Expression levels of imprinted genes *ATCDC48 *and *PDE120 *are regulated by methylation pathways

**Figure 5:**
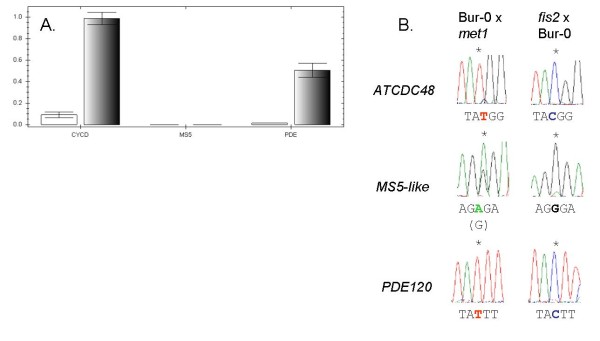
**Response of imprinted the *ATCDC48*, *PDE120 *and *MS5-like *loci to mutation of *MET1***. **5A**. The imprinted loci *ATCDC48 *(left-hand columns) and *PDE120 *(right-hand columns), which are associated with DMRs, are aberrantly upregulated in rosette tissue of *met1-3 *mutant plants whilst *MS5-like *remains unexpressed (middle columns). In each case the Col-0 wild-type expression is shown on the left in white, *met1-3 *expression in black on the right. Values were derived as the means of four biological replicates; standard errors are shown. **5B**. *MET1 *is not required for repression of the paternal alleles of *ATCDC48*, *PDE120 *or *MS5-like *(left-hand column); *FIS2 *is not required for uniparental expression of the maternal alleles (right-hand column).

In order to confirm whether DNA methylation changes are associated with altered expression levels of our novel imprinted genes, we performed qRT-PCR on cDNA derived from seedlings of *met1-3 *plants and found that there is a significant aberrant induction of the imprinted MEGs *ATCDC48A *and *PDE120 *in *met1-3 *mutants (Figure 5A). In concordance with the failure to detect a candidate DMR for *MS5-like*, no such induction occurred for this gene (Figure 5A). Interestingly, however, seeds generated by fertilising wild-type *Arabidopsis thaliana *with pollen from *met1-3 *plants did not cause a reactivation of the paternal allele of any of the three genes (Figure 5B). The maternal FIS-complex has also been recently shown to regulate imprinting of certain MEGs [37,44-46]. However, for the three imprinted loci of focus in this study, we found that fertilising *fis2 *plants with wild-type pollen did not lead to any loss of imprinting either (Figure 5B). Overall, this could imply that the proximal DMRs we have identified do not function as ICRs for these imprinted loci. Alternatively, it may suggest the existence of a subset of imprinted MEGs in which imprinted status and expression levels are regulated via a *MET1*- and *DME/FIS*-independent pathway. The lack of response of these three genes to these epigenetic modifier pathways offers a further explanation for the failure of Hsieh et al. [24] to detect *ATCDC48A, MS5-like *and *PDE120 *as imprinted MEGs, as their filtering approach compared numbers of sequence reads in wild-type crosses with those crossed to such epigenetic modifier backgrounds.

## Discussion

In comparison with current knowledge of genomic imprinting (i.e. regarding number of imprinted genes and regulatory mechanisms) in mammalian genomes, the study of genomic imprinting in plants has been hindered by the low number of imprinted genes that have been reported and studied to date. In this study, we have sought to address this by identifying novel imprinted genes in the model plant *Arabidopsis thaliana *and considering our results in the light of screens performed by others, and of current theories concerning the regulation of imprinting in plants.

In this study, we have conducted a genome-wide allele-specific expression analysis screen using cDNA-AFLP to identify 93 maternally expressed TDFs from a total of 4500 polymorphic allele-specific TDFs. Some of these may represent candidate maternally expressed genes regulated by imprinting in the model plant *Arabidopsis thaliana*. To identify the genes represented by each TDF, we developed a novel bioinformatics software program called GenFrag which can directly identify genes (in well annotated sequenced genomes e.g. Col-0 accession) based only on the size of the TDF and the selective nucleotides of the primers used to generate the TDF. Although cDNA-AFLP is an early generation transcriptomics platform, as a technique it has some distinct advantages over probe hybridisation based approaches such as microarrays. These advantages include: (a) applicability to any species (including species with no genomic information), (b) low cost and reproducibility, (c) small amounts of RNA template needed, (d) detection of lowly expressed genes and (e) high specificity to distinguish closely related genes [47-50]. However, one of the most time-consuming steps in the cDNA-AFLP technique is the excision of TDFs from gels so that the TDF can be sequenced (typically following amplification and/or subcloning into a plasmid). To increase the throughput of gene identification in cDNA-AFLP experiments involving species with sequenced and well annotated genomes (such as *Arabidopsis thaliana*), we developed the GenFrag bioinformatics software program.

There have been previous efforts to develop bioinformatic approaches to improve the efficiency of (cDNA-)AFLP techniques. The large amount of DNA sequence data available for several species has been used for *in silico *predictions of virtual transcript profiles. Tailor-made software, such as *AFLPinSilico *[51] and GenEST [52,53], allow high-throughput identification of AFLP and cDNA-AFLP TDFs for *Arabidopsis thaliana *and *Globodera rostochiensis*, respectively. These *in silico *approaches were also developed to enable experiment simulations, decreasing the time needed for AFLP optimisation, and the number of samples which need to be processed [51-53]. The GenFrag program developed in this study is designed to facilitate high throughput direct identification of genes from cDNA-AFLP experiments with fully sequenced well-annotated genomes such as that of *Arabidopsis thaliana*. We have made the GenFrag program freely available to the research community at: http://www.nem.wur.nl/UK/Research/bio/.

In our study to identify novel imprinted genes in *Arabidopsis thaliana*, we applied the GenFrag program to the 93 TDFs displaying a maternal-specific expression pattern, and could thereby identify 52 maternally expressed genes (MEGs) in *Arabidopsis thaliana *(Table 1). By filtering for expression within seeds and enrichment within endosperm tissues, we ranked 18 MEGs on the basis of the absolute difference of their expression levels between the seed coat and the endosperm (Table 2). The identification of *MS5-like *and *PDE120 *was also supported by alternative approaches i.e. comparison with the dataset of Day et al. ([36]; Table 1) and ranking by ratio of Endosperm/Seed Coat expression (Additional file 6 Table S5). For any given gene expressed in the developing seed, it is difficult to separate both the absolute and relative contributions of the different seed tissues, especially given their differing ploidies (triploid in the endosperm, diploid maternal in the seed coat, diploid hybrid in the embryo) and the differences in cellular/nuclear abundance for the different tissues (seed coat, endosperm, embryo). As the contributions to total transcription are normalised against units of RNA no direct determination of the absolute contributions from each seed tissue is possible. However, we can demonstrate that biallelic expression in the seed is detectable at the developmental stage we sample through use of a biallelic endosperm expressed gene (*PHE2*) as a positive control (Table 3). Our approach does have the advantage of allowing a focus on highly expressed genes, whose transcripts in seeds 4 dap are least likely to have been maternally deposited in the central cell prior to fertilisation. The endosperm is transcriptionally active immediately following fertilization, such that maternally deposited, long-lived RNAs are unlikely to play an important role [34] or be found at high levels in endosperm tissues 4 dap. This contrasts with the early development of the embryo, where expression in the embryo is maternally-biased (88% of transcripts at the 2-4 cell stage, for example), with paternal alleles subsequently becoming reactivated at the later globular stages of embryo development [32]. Hence, the top ranked endosperm-enriched genes identified in our study can be considered to be the most likely imprinted genes (Table 2).

A striking finding in our study is that there is little overlap in terms of genes detected between all of the different screens for imprinted genes in *Arabidopsis thaliana *conducted to date, including our study (Additional file 10 Figure S5). Possible explanations for such lack of overlap can include (a) use of different accessions (genetic backgrounds); (b) use of samples from different developmental stages (where the relative abundance and contribution of embryo versus endosperm tissues will differ); (c) use of different filtering criteria; (d) use of different experimental approaches for isolation of seed, embryo and endosperm tissues and RNA from each tissue; and (e) use of different transcriptome profiling platforms and bioinformatic pipelines. In this study we demonstrate that the imprinted genes we have identified are unlikely to be detected at the later developmental stage used by Hsieh et al. [24], whilst the lack of overlap between the next-generation sequencing approaches of Hsieh et al. (2011) and Wolff et al. [23] is likely contributed to the analysis of different time points (7-8 DAP *versus *4 DAP) and different accessions (Col-0 × Ler*-*0 *versus *Col-0 × Bur-0). There is some overlap (7 genes) between the RNA sequencing approach of [23] (Col-0 × Bur-0 crosses) and a screen for genes regulated by DMRs in Col-*gl *X L*er*-0 crosses [25] suggesting that DMRs may control gene-specific imprinting for a limited number of loci, and/or that their ability to do so may vary according to different genetic backgrounds. Although it seems likely that all these approaches have identified imprinted genes it would seem that detection of imprinted loci (gene-specific or allele-specific) may be dependent upon accessions (genetic backgrounds), developmental stages sampled and experimental methodology. These factors may introduce significant variation between the results of different studies. Given the increasing numbers of allele-specific expression effects being detected in plants, it may be opportune for the imprinting research community to develop some common standards for the definition and validation of imprinted genes in flowering plants (see also [11]).

For the top three ranked genes *ATCDC48, PDE120 *and *MS5-like*, using LCM, we could independently detect expression of these genes in 4 dap seed tissues (seed coat, endosperm and embryo) (Additional file 7 Figure S2). For *ATCDC48 *and *PDE120 *we also confirmed that expression was low in pre-fertilized ovules but increased during the course of seed development (Figure 1A, B), which is consistent with these genes being subject to post-fertilisation expression in the developing seed (i.e. not maternally deposited). We confirmed that all three of these endosperm-expressed genes are maternally expressed in 4 dap reciprocal F1 hybrid seeds from different accessions and hence represent novel cases of gene-specific imprinting in *Arabidopsis thaliana *(Figures 2 and 3). While *ATCDC48 *and *PDE120 *are subject to binary imprinted expression, *MS5-like *shows a preferential maternal expression pattern of imprinting [9,21], as some paternal expression is also detected (Figure 2). Although the expression levels of *MS5-like *were similar in Col-0 and L*er*-0 (Figure 1), and in the pattern determined for Ws-0 (Seed Genes Network), the extent of imprinting did vary, with the C24 and Bur-0 alleles displaying a greater extent of imprinting when paternally inherited.

ICRs of imprinted genes often overlap with DMRs. Hence, we considered that our top-ranked imprinted genes *ATCDC48*, *PDE120 *and *MS5-like *might contain candidate DMRs in their genomic vicinity and that, if so, these could be candidate ICRs. We could identify DMRs upstream of *PDE120 *and one DMR downstream of *ATCDC48 *that could potentially act as ICRs (Figures 4A and 4B). However, the difference in methylation between wild-type and *dme *endosperm did not reveal any DMR for *MS5-like *(Figure 4C). Expression of *DME *in the central cell leads to hypomethylation of the maternal genome. However, the methylation data used [43] represent the global methylation status of both the maternal and paternal genomes of the endosperm. This could explain why no DMR could be identified for *MS5-like*. Control of imprinting at the *MS5-like *locus may be independent of DNA methylation, or be regulated by a DMR far distal to the gene. Methylation-independent imprinting has been observed for some imprinted loci in mammals [54] and histone methylation by *Polycomb *group proteins has been shown to regulate several imprinted genes in plants [37,44,55]. Our results indicate that lack of *MET1 *in the male gamete has no effect on imprinting of *ATCDC48, PDE120 *and *MS5-like *in the developing seed. In contrast, we find that lack of *MET1 *leads to overexpression of *ATCDC48 *and *PDE120 *in vegetative leaf tissues. No effects of lack of *MET1 *in vegetative tissues were observed for *MS5-like*. Taking into consideration the recent findings of [23] and previous reports showing that PcG complexes regulate imprinting [37,44-46], we also tested for possible effects of the maternal FIS-complex on regulation of the three maternally expressed imprinted genes and found that fertilising *fis2 *plants with wild-type pollen did not lead to any loss of imprinting. Hence, alternative epigenetic pathways are likely to regulate imprinting of *MS5-like*. Such regulation can neither be ruled out for *ATCDC48 *and *PDE120*. Further characterization of the imprinted *ATCDC48*, *PDE120 *and *MS5-like *loci will provide opportunities for increasing our understanding of the epigenetic mechanisms involved in the regulation of genomic imprinting in angiosperms.

The maternally expressed imprinted gene, *ATCDC48A*, is a homohexameric AAA(+) ATPase chaperone implicated in cell cycle control and cell proliferation. CDC48/p97 represents a highly conserved protein which plays a role as an initiation factor for DNA replication in many species [56] and has been shown to be essential in a wide range of multicellular and unicellular organisms [57]. In plants, the CDC48A protein has been shown to physically interact with the SOMATIC EMBRYOGENESIS RECEPTOR LIKE KINASE 1 (SERK1) protein [58,59]. The *Arabidopsis thaliana *genome contains three CDC48 loci, *ATCDC48A *(At3g09840), *ATCDC48B *(At3g53230) and *ATCDC48C *(At5g03340). *ATCDC48A *can functionally complement CDC48 mutants of *Saccharomyces cerevisiae *[56], and loss of the *PUX1 *negative regulator of *ATCDC48 *leads to accelerated plant growth due to increased cell division and expansion [60]. Additional studies in *Arabidopsis thaliana *conducted with T-DNA knockout lines of *AtCDC48A *have demonstrated that homozygous null seedlings are viable until 5 days old but die shortly thereafter. It was also demonstrated that null *Atcdc48a *alleles have a drastically reduced transmission efficiency through the male gametophyte (i.e. *ATCDC48A *is essential for normal pollen germination and tube elongation) [57].

Our results indicate that *ATCDC48A *is maternally expressed and subject to genomic imprinting in the developing seed (endosperm) (Figures 1, 2 and 3). Although the imprinting status of the maize homolog of *ATCDC48A *has not yet been determined, it is possible that imprinting of the maize homolog of *ATCDC48A *(or other cell-cycle genes) could be responsible for the dosage effects on cell-cycle progression observed in endosperm from interploidy crosses of maize [61]. While a clear role for *ATCDC48 *in the control of DNA replication in plant cells has not yet been established, our findings that *ATCDC48 *is a maternally expressed imprinted gene in developing endosperm resonates with a role in controlling proliferation as suggested for imprinted genes by the parental conflict theory [12].

Less is known from a functional perspective regarding the other two imprinted genes identified in this study. The *MS5-like *maternally expressed imprinted gene has sequence similarity to Male Sterile 5 (*MS5*), a gene that has been shown to be essential for male meiosis in *Arabidopsis thaliana *[62]. *MS5-like *also displays sequence similarity with the sulphur deficiency-induced gene *AtSDI1 *[63].

The maternally expressed imprinted gene *PDE120 *is annotated as a *pigment defective embryo *(*pde*) mutant in the SeedGenes database [64,65]. The nuclear encoded *PDE120 *locus encodes the TIC40 protein which is a component of the protein import apparatus of the inner envelope of the chloroplast [66]. The identification of a maternally expressed imprinted nuclear gene which encodes a protein product targeted to the maternally-inherited chloroplasts could be suggestive of selection for imprinting at nuclear loci where strong control by maternally-inherited alleles of chloroplast function is essential [67].

## Conclusions

In this study we have identified 52 maternally expressed genes in siliques containing reciprocal F1 hybrid seeds. We have developed and employed a novel bioinformatics tool called GenFrag to facilitate higher-throughput analysis of cDNA-AFLP experiments on organisms with well-annotated sequenced genomes. We ranked the 52 maternally expressed genes according to their relative expression levels in the endosperm *versus *seed coat tissues at the globular embryo stage and chose the three top-ranked imprinted candidate genes for further investigation. We confirmed expression of the three candidates in 4 dap seeds by LCM RT-PCR and further confirmed maternal-specific expression of the three genes in 4 dap F1 hybrid seeds generated with different *Arabidopsis thaliana *accessions. Taken together, our results indicate that *ATCDC48 *is a maternally expressed imprinted gene in the developing *Arabidopsis thaliana *seed, and is likely imprinted in the endosperm and perhaps the embryo. Confirmation of imprinted maternal expression was also demonstrated for the other two top-ranked genes *PDE120 *and *MS5-like*. Where present, DMRs for each of the three imprinted genes and the 18 maternally expressed genes in Table 2 were identified and posited as putative ICRs. However, analysis of the imprinted *ATCDC48*, *PDE120 *and *MS5-like *loci with the candidate modifiers *met1-3 *and *fis2 *indicates that the regulation of imprinting at these three genes is independent of DNA methylation and the FIS-complex. Overall, our study identifies novel maternally expressed genes in *Arabidopsis thaliana *seed and validates three genes (*ATCDC48*, *PDE120 *and *MS5-like*) as novel maternally expressed imprinted genes in *Arabidopsis thaliana *seed. Further analysis of the genes identified here and by others will accelerate efforts to increase our understanding of the epigenetic regulatory mechanisms and evolution of imprinted genes in flowering plants.

## Methods

### Plant growth and generation of cDNA

*Arabidopsis thaliana *L. of accessions Col-0, L*er*-0, C24 and Bur-0 were grown on 8 parts Westland multipurpose compost (Dungannon, N. Ireland): 1 part perlite: 1 part vermiculite under the following conditions: 200 μmol m^-2 ^s^-1 ^at 21°C/18°C and a 16:8 hr light:dark cycle. F1 hybrid seeds were generated via reciprocal crosses of Col-0 and Ler-0, Bur-0 and C24 accessions [24,25]. Plants were manually emasculated before anthesis and reciprocally crossed by hand under a Leica MZ6 dissecting microscope (Leica Microsystems CMS GmbH, Ernst-Leitz-Straße 17-37, Wetzlar, D-35578, Germany) using Dumostar No. 5 tweezers (Dumont Biology, Switzerland). Siliques and seeds were harvested at the time points described. mRNA was extracted in combination with on-column DNase treatment using an RNase-free DNase kit (Qiagen, USA). 5 μg of total RNA were hybridized to biotinylated oligo dT which binds the streptavidin-coated PCR tube wall (mRNA Capture Kit, Roche) and cDNA synthesis performed (Quantitect Reverse Transcriptase kit, Qiagen). Quality control was performed on the Agilent 2100 Bioanalyzer (Agilent Technologies Schweiz AG, Basel, Switzerland). Samples were stored at -80°C prior to use.

### cDNA-AFLP

cDNA from siliques was generated as described, digested with restriction enzymes *Bst*YI and *Mse*I and ligated with adapters complementary to the restriction site of *Bst*YI (5'-CTCGTAGACTGCGTAGTGATCYGATCCGTTCA-3 and 3'-CATCTGACGCATCACTAGRCTAGGCAAGT-5) and *Mse*I (5'-GACGATGAGTCCTGAGTAACACTGGATCATG-3' and 3'-CTACTCAGGACTCATTGTGAGGTAGTAC-5). The ligated fragments were selectively amplified a first time using *Mse*I primer (5'-GATGAGTCCTGAGTAA-3') and *Bst*YI primers (5'-GACTGCGTAGTGATCCN-3 and 5'-GACTGCGTAGTGATCTN-3'). The amplified fragments were diluted 1:20 and amplified a second time using 128 primer combinations (8 *Bst*YI possible primers 5'-GACTGCGTAGTGATCCNN-3 and 5'-GACTGCGTAGTGATCTNN-3' × 16 *Mse*I possible primers 5'-GATGAGTCCTGAGTAANN-3' = 128 combinations). Products were run on polyacrylamide gels and visualised with the GelDoc-ItTM Imaging System (Ultra-Violet Products Ltd., Cambridge, UK). Samples were processed using the 16-capillary 3130 × l Genetic Analyser (Applied Biosystems Inc.). 0.5 μl reaction products were mixed with 0.4 μl Internal Lane Standard 600 ROXTM size standard (Promega, WI, USA) or GeneScanTM 500 ROXTM size standard (Applied Biosystems, UK), in 9 μl Hi-Di Formamide (Applied Biosystems, UK). Fragments were analysed in a multiplex run and visualised with *Bst*YI+C and *Bst*YI+T primers, respectively labelled with the fluorescent dyes JOE and 6-FAM. Samples were analysed using the GeneMapper v3.7 software, which assigned each TDF an allelic label, or bin, based on its size as determined by comparison to the ILS600-C marker (Promega). Bin assignment permitted a variation of ± 0.5 bp in the determined size. For cDNA-AFLP samples generated with a given primer combination, the two parental lines, Col-0 × Col-0 and L*er*-0 × L*er*-0, and the two reciprocal hybrids, Col-0 × L*er*-0 and L*er*-0 × Col-0 were analysed together within a run to allow identification of polymorphic and differentially expressed TDFs. Fragment-sizing and allele-calling parameters for GeneMapper were normalized to the data using the default Sum-of-Signal method; alleles common between samples were not deleted. This generated electropherograms matching detected peaks with their allele calls, from which genotypes were derived.

### Development of GenFrag program & software

We downloaded the two datasets containing the available full-length *Arabidopsis thaliana *cDNAs from the TIGR v.4.0 (released March 2005) and TAIR v.7 databases at ftp://ftp.tigr.org/pub/data/a_thaliana/ath1/SEQUENCES/ and ftp://ftp.arabidopsis.org/home/tair/Sequences/ (released April 2007) respectively. *Arabidopsis thaliana *ESTs were downloaded fromhttp://www.plantgdb.org/download/Download/Sequence/ESTcontig/Arabidopsis_thaliana/current_version/Arabidopsis_thaliana.mRNA.PUT.fasta.bz2 and a dataset of alternative splicing variants from TIGR-Atg database (release June 2003) at http://www.tigr.org/tdb/e2k1/ath1/altsplicing/splicing_variations.shtml.

GenFrag expands on the earlier GenEst package [53] by providing a web interface which is publicly available at the URL: http://www.nem.wur.nl/UK/Research/bio/. GenFrag provides full named support for all known restriction enzymes as listed in REBASE [68], additional support for primer combinations, their size corrections, and a listing of mismatched fragment sizes. GenFrag also allows a subset of experimental allelic fragments to be selected for analysis on the basis of the potential interest of genes in a candidate sequence list i.e. rather than sequencing all fragments. The GenFrag software is written in Ruby, and can be run on all platforms supported by Ruby, including Windows, OSX, Linux and the Java virtual machine. The restriction enzyme module is available as part of the Open Bioinformatics Foundation BioRuby toolkit [69] and includes all known restriction enzymes by name. Genomic information can be read in any BioRuby supported format, including FASTA. The web interface is written in Ruby on Rails, and SQLite is used for caching searches. GenFrag software can be used in two ways: through a public web interface and as a software module in a computing pipeline.

### Expression analysis

Microarray data of gene expression levels and absence calls from Seedgenenetwork (Harada-Goldberg Arabidopsis Laser Capture Microdissection Gene Chip Data Set, http://seedgenenetwork.net) were downloaded from Gene Expression Omnibus [70], accession numbers GSM284397 and GSM284398 (seed coat), GSM284390 and GSM284391 (peripheral endosperm), GSM284388 and GSM284389 (micropylar endosperm), GSM284392, GSM284393 and GSM284394 (chalazal endosperm) and GSM284384 and GSM284385 (embryo). The developmental stage sampled by these experiments is the globular stage of embryo development. The mean expression value of all replicates was used. The following genes did not have probes: At1g12420, At1g55320, At2g45315, At3g21465, At4g01000, At4g25315, At5g04895, At5g35737 and At5g40240. Probes for At4g37530 and At1g14880 also matched another gene so were omitted from the analysis due to the possibility of ambiguous results.

### LCM

Siliques of emasculated and hand-crossed plants of accession L*er*-0 were collected and directly transferred to an ASP200 embedding machine (Leica Microsystems GmbH, Wetzlar, Germany) and dehydrated at room temperature in a graded ethanol series (1 hour at 70%, 3 × 1 hour at 90%, 3 × 1 hour at 99.98%) and in xylol (2 × 1 hour and 1 × 75 minutes) which was substituted by Paraplast X-tra embedding media (Roth AG, Arlesheim, Switzerland) at 56°C (2 × 1 hour, 1 × 3 hours), poured into paraffin blocks and stored at 4°C. Paraffin blocks were cut to 10 μm thin sections on an RM2145 microtome (Leica Microsystems GmbH, Wetzlar, Germany) and mounted on nuclease-free membranes held in metal frame slides in methanol, dried overnight at 42°C and deparaffinised in xylol at 56°C (3 × 10 minutes). Microdissection was performed on thin sections of siliques using the MMI CellCut Plus laser capture microscope (MMI Molecular Machines and Industries AG, Glattburg, Switzerland) to generate *circa *150 cuts (1500 cells) per sample. Total RNA was extracted from pooled samples using the PicoPure RNA isolation kit (Arcturus Engineering, Mountain View, CA 94043-4019, USA) and single-stranded cDNA generated using the NuGEN WT-Ovation Pico RNA Amplification System (NuGEN Technologies Inc., Brockville, Canada).

### RT-PCR

Primers for the three top ranked candidate genes were designed using the Universal ProbeLibrary Assay Design Center (Roche, Switzerland, http://www.roche-applied-science.com) Identical PCR conditions were used for all genes, with Tm of 59°C and 40 amplification cycles. Two replicates were performed (data not shown), one representative result is shown for the three top ranked candidate imprinted genes analysed (Additional file 7 Figure S2). Quantitative RT-PCR was performed on biological triplicate samples using SYBR Green master mix (ABI) and run on a C1000 Thermal CycLer incorporating the CFX Real-Time System. Details of all primers are available on request.

### DNA sequencing & QUASEP

Exonic SNPs between *Arabidopsis thaliana *accessions were identified at The Arabidopsis Information Resource [71] (PERL0437780 for *ATCDC48*, PERL0895299 for *PDE120*, PERL0626585 for *MS5-like *and Exon 2, 2345566 (C/T) for *PHE1*). cDNA from seeds of reciprocal Col-0 × C24 and Col-0 × Ler-0 crosses was generated as described. Sequences surrounding the SNPs were amplified by PCR performed under standard conditions with GoTaq (Invitrogen) and sequenced by GATC. Quantification of maternally- and paternally-derived SNPs was performed via QUASEP (Quantification of Allele-Specific Expression by Pyrosequencing). RT-PCR was performed with Quantitect RT kits according to manufacturer's instructions. PCR was performed on cDNA using one biotinylated primer per pair using sequences adapted from assays designed by PSQ assay software (sequences available on request). Mean values of parental expression were calculated from at least three replicates. Genomic DNA and the genes *FWA*, *PHE1 *and *PHE2 *were used as controls.

### Identification of DMRs

High-throughput bisulfite sequencing data of *Arabidopsis thaliana *wild-type endosperm and endosperm from seeds deficient for a maternal DME allele [43] were retrieved from ArrayExpress (http://www.ebi.ac.uk/arrayexpress, accession number E-GEOD-15922), corresponding to the TAIR 8 version of the genome. The percentage of methylation at cytosines situated between the genes immediately upstream and downstream of our candidates was calculated. Regions that showed a difference between *dme *and wild-type endosperm cytosine methylation percentages were identified as DMRs and potential ICRs.

## Authors' contributions

PMcK designed assays, performed sequencing and pyrosequencing, and prepared the manuscript. SLD performed the cDNA-AFLP screen, analyzed TDF data, and performed pyrosequencing controls. PP contributed *in-silico *cDNA-AFLP and, with TJW, developed the GenFrag software. PW generated cDNA sequence traces from Col-0 × Bur-0 accessions and crosses to mutant modifiers. MS performed LCM and RT-PCR experiments. MTAD analysed and compared data-sets, determined expression ratios of candidate genes, prepared figure S1 and formatted and edited the manuscript. AF and DD performed qRT-PCR. NTL generated hybrid cDNA and conducted sequencing reactions. AC identified differentially methylated regions and edited the manuscript. TJW and GS supervised the development of GenFrag. CK and UG assisted experimental design, UG supervised and funded the performance of LCM and related expression analyses. CS designed experiments, raised financing for their implementation, oversaw the experiments and development of the project, and prepared the final manuscript. All authors read and confirmed the manuscript.

## Supplementary Material

Additional file 1 **Table S1 - Known imprinted genes in flowering plants**. In the angiosperms, eleven imprinted genes had been reported from *Arabidopsis thaliana *and related species, six from maize and one from rice. All but three are expressed solely from the maternally inherited allele. With the exception of *MEDEA*, for which conflicting reports have been published (for discussion see [72]), all imprinted *Arabidopsis thaliana *genes show mono-allelic expression only in the terminally differentiating endosperm, whereas maize *Mee1 *clearly shows imprinted expression in the maize embryo. Click here for file

Additional file 2 **Figure S1 - Flow chart of the GenFrag program**. Generation of the Transcript Derived Fragment (TDF) by poly-A capture of mRNA from silique tissue, followed by two digestions using first *Bst*YI then *Mse*I restriction enzymes. The fragments were ligated to adapters and selectively amplified using the primer combinations described (Additional file 4, **Table S3**). Transcript sizes were then determined on the ABI3130xl Genetic Analyzer. TDFs which were called as present in the maternal hybrid cross direction but absence from the reciprocal cross were considered as being derived from candidate MEGs (Additional file 3, **Table S2**). Subsequently, transcript sequences were assigned to progenitor genes by GenFrag using publicly available Arabidopsis transcript sequences. The transcript goes through two *in silico *digestions using first *Bst*YI then *Mse*I restriction enzymes to produce one fragment per transcript mimicking the *in vitro *protocol from which the gene identify is determined (Table 1). Click here for file

Additional file 3 **Table S2 - Relative proportions of uniparental TDFs expressed in siliques as determined by cDNA-AFLP of hybrid Col-0 × Ler-0 reciprocal crosses across three timepoints**. Click here for file

Additional file 4 **Table S3 - Identification of 93 TDFs showing maternal-specific expression using the GenFrag software**. 93 TDFs showing maternal-specific expression were analysed by GenFrag. Gene identitites were predicted using combinations of primers designed against *Bst*YI and *Mse*I cutting sites (3^rd ^and 4^th ^columns) and the size of the TDF as determined by capillary electrophoresis (5^th ^column) as unique identifiers. Single unique genes were predicted for 52 TDFs out of the 93 maternal-specific TDFs (7^th ^column, TDFs 1-52). A further 21 TDFs (TDFs 53-73) rendered non-unique predictions and 20 others (TDFs 74-93) could not be matched by GenFrag to any known *Arabidopsis thaliana *sequence. For 8 TDFs, the predicted size (marked with *) differed from that determined by capillary electrophoresis, by amounts indicated in parentheses. Click here for file

Additional file 5 **Table S4 - Lack of detection of known imprinted genes is due to lack of SNPs in restriction sites**. MseI cuts in the T/TAA context, Bst YI cuts in the R/GATCY context (source: SALK SNP viewer, TAIR). Click here for file

Additional file 6 **Table S5 - Relative expression levels of maternally-expressed seed genes in the endosperm and seed coat**. Genes detected as maternal by cDNA-AFLP with a log2-ratio of higher than 1 (indicating expression twice as high in endosperm vs. seed coat) are listed. Click here for file

Additional file 7 **Figure S2 - Analysis of expression profiles of *ATCDC48*, *PDE120 *and *MS5-like *in LCM tissues of *Arabidopsis thaliana *seeds (4 dap)**. Results of RT-PCR performed on cDNA derived from LCM endosperm (ES), seed coat (SC) and embryo (EM) tissues, shown for one representative replicate of two. *ACT11 *and *UBC9 *were used as loading controls. Click here for file

Additional file 8 **Figure S3 - Confirmation of maternal expression of cDNA-AFLP candidate genes in crosses to Ler-0**. cDNA from 4 dap tissue was amplified and sequenced from F1 L*er*-0 × Col-0 hybrid seeds and *MS5-like *and *PDE120 *found to be maternally expressed. Click here for file

Additional file 9 **Figure S4 - Confirmation of six further cDNA-AFLP genes as maternal in seed tissue**. cDNA from F1 4 dap seed tissue was amplified and sequenced. In each case, cDNA from Col-0 × C24 is shown on the left, C24 × Col-0 is shown on the right. SNPs are marked with asterisks. Click here for file

Additional file 10 **Figure S5 - Divergent candidate imprinted genes identified by different screens in Arabidopsis**. The overlap between maternally-expressed genes identified from this cDNA-AFLP screen performed in 3-5 dap Col-0 × L*er*-0 crosses (red); those predicted from analysis of DMRs identified from Col-0 × L*er*-0 endosperm (blue, [25]) and those identified by next generation screening approaches ([24] yellow; [23]; green) (see descriptions in Introduction). Click here for file

Additional file 11 **Figure S5 - Identification of DMRs located in the vicinity of candidate imprinted genes (Table **2**) and other maternally inherited genes**. Difference in methylation percentage between *dme *and wild-type endosperm for cytosine bases in the vicinity of three candidate imprinted genes (Table 2). Grey boxes represent the gene body in a 5'-3' orientation, red bars highlight DMRs. Click here for file
