# Supplementary material for: Identification of imprinted genes subject to parent-of-origin specific expression in Arabidopsis thaliana seeds
Source: BMC Plant Biol. 2011 Aug 12;11:113. doi: 10.1186/1471-2229-11-113 (PMC3174879; doi:10.1186/1471-2229-11-113)
Supplement: Additional file 1 — Table S1 - Known imprinted genes in flowering plants. In the angiosperms, eleven imprinted genes had been reported from Arabidopsis thaliana and related species, six from maize and one from rice. All but three are expressed solely from the maternally inherited allele. With the exception of MEDEA, for which conflicting reports have been published (for discussion see [72]), all imprinted Arabidopsis thaliana genes show mono-allelic expression only in the terminally differentiating endosperm, whereas maize Mee1 clearly shows imprinted expression in the maize embryo. [file 1471-2229-11-113-S1.DOC]

| **Gene** | **Organism** | **Maternally or paternally expressed imprinted gene?** | **Reference** |
| --- | --- | --- | --- |
| *MEDEA* | Arabidopsis | Maternally | Vielle-Calzada et al., 1999 |
| *FIS2* | Arabidopsis | Maternally | Jullien et al., 2006 |
| *PHE1* | Arabidopsis | Paternally | Köhler et al., 2004 |
| *FWA* | Arabidopsis | Maternally | Kinoshita et al., 2004 |
| *MPC* | Arabidopsis | Maternally | Tiwari et al., 2008 |
| *HDG3* | Arabidopsis | Paternally | Gehring et al., 2009 |
| *HDG8* | Arabidopsis | Maternally | Gehring et al., 2009 |
| *HDG9* | Arabidopsis | Maternally | Gehring et al., 2009 |
| At5g62110 | Arabidopsis | Maternally | Gehring et al., 2009 |
| At*MYB3R2* | Arabidopsis | Paternally | Gehring et al., 2009 |
| At*FH5* | Arabidopsis | Maternally | Gerald et al., 2009 |
| Os*FIE* | Rice | Maternally | Luo et al., 2009 |
| *Npr1* | Maize | Maternally | Guo et al., 2003 |
| *Meg1* | Maize | Maternally | Gutierrez-Marcos et al., 2004 |
| *Fie1* | Maize | Maternally | Gutierrez-Marcos et al., 2006 |
| *Fie2* | Maize | Maternally | Gutierrez-Marcos et al., 2006 |
| *Mez1* | Maize | Maternally | Haun et al., 2007 |
| *Mee1* | Maize | Maternally | Jahnke and Scholten, 2009 |
