# Supplementary material for: Identification of imprinted genes subject to parent-of-origin specific expression in Arabidopsis thaliana seeds
Source: BMC Plant Biol. 2011 Aug 12;11:113. doi: 10.1186/1471-2229-11-113 (PMC3174879; doi:10.1186/1471-2229-11-113)
Supplement: Additional file 2 — Figure S1 - Flow chart of the GenFrag program. Generation of the Transcript Derived Fragment (TDF) by poly-A capture of mRNA from silique tissue, followed by two digestions using first BstYI then MseI restriction enzymes. The fragments were ligated to adapters and selectively amplified using the primer combinations described (Additional file 4, Table S3). Transcript sizes were then determined on the ABI3130xl Genetic Analyzer. TDFs which were called as present in the maternal hybrid cross direction but absence from the reciprocal cross were considered as being derived from candidate MEGs (Additional file 3, Table S2). Subsequently, transcript sequences were assigned to progenitor genes by GenFrag using publicly available Arabidopsis transcript sequences. The transcript goes through two in silico digestions using first BstYI then MseI restriction enzymes to produce one fragment per transcript mimicking the in vitro protocol from which the gene identify is determined (Table 1). [file 1471-2229-11-113-S2.PDF]

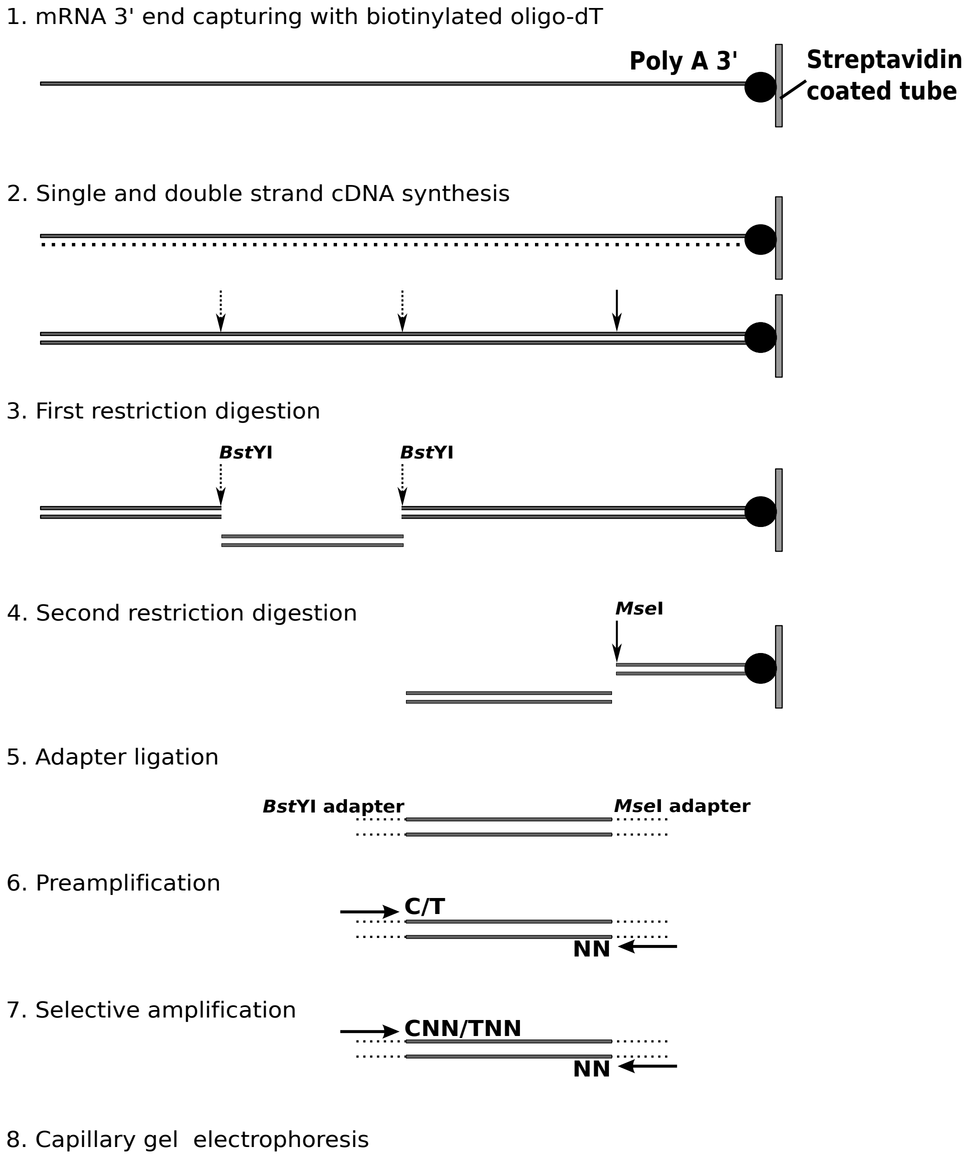
1. mRNA 3' end capturing with biotinylated oligo-dT
Poly A 3'
Streptavidin coated tube
2. Single and double strand cDNA synthesis
3. First restriction digestion
BstYI
BstYI
4. Second restriction digestion
MseI
5. Adapter ligation
BstYI adapter
MseI adapter
6. Preamplification
C/T
NN
7. Selective amplification
CNN/TNN
NN
8. Capillary gel electrophoresis
