# Supplementary material for: Identification of imprinted genes subject to parent-of-origin specific expression in Arabidopsis thaliana seeds
Source: BMC Plant Biol. 2011 Aug 12;11:113. doi: 10.1186/1471-2229-11-113 (PMC3174879; doi:10.1186/1471-2229-11-113)
Supplement: Additional file 3 — Table S2 - Relative proportions of uniparental TDFs expressed in siliques as determined by cDNA-AFLP of hybrid Col-0 × Ler-0 reciprocal crosses across three timepoints. [file 1471-2229-11-113-S3.DOC]

|  | **3 dap** | **4 dap** | **5 dap** |
| --- | --- | --- | --- |
| Total TDFs | 2842 | 2724 | 2187 |
| Number of maternally inherited Col-0 alleles (TDFs) | 315 | 366 | 403 |
| Number of maternally inherited L*er*-0 alleles (TDFs) | 505 | 306 | 355 |
