# Supplementary material for: Identification of imprinted genes subject to parent-of-origin specific expression in Arabidopsis thaliana seeds
Source: BMC Plant Biol. 2011 Aug 12;11:113. doi: 10.1186/1471-2229-11-113 (PMC3174879; doi:10.1186/1471-2229-11-113)
Supplement: Additional file 4 — Table S3 - Identification of 93 TDFs showing maternal-specific expression using the GenFrag software. 93 TDFs showing maternal-specific expression were analysed by GenFrag. Gene identitites were predicted using combinations of primers designed against BstYI and MseI cutting sites (3rd and 4th columns) and the size of the TDF as determined by capillary electrophoresis (5th column) as unique identifiers. Single unique genes were predicted for 52 TDFs out of the 93 maternal-specific TDFs (7th column, TDFs 1-52). A further 21 TDFs (TDFs 53-73) rendered non-unique predictions and 20 others (TDFs 74-93) could not be matched by GenFrag to any known Arabidopsis thaliana sequence. For 8 TDFs, the predicted size (marked with *) differed from that determined by capillary electrophoresis, by amounts indicated in parentheses. [file 1471-2229-11-113-S4.DOC]

|  | ***Bst*YI primer** | ***Mse*I primer** | **TDF (determined)** | **TDF (predicted)** | **Gene ID** |
| --- | --- | --- | --- | --- | --- |
| 1 | *Bst*YI-T2 | *Mse*I-1 | 228.72 | 229 | At1g03070 |
| 2 | *Bst*YI-T4 | *Mse*I-13 | 100.3 | 100 | At1g04700 |
| 3 | *Bst*YI-C3 | *Mse*I-12 | 179.65 | 180 | At1g10000 |
| 4 | *Bst*YI-T2 | *Mse*I-13 | 84.5 | 84 | At1g12420 |
| 5 | *Bst*YI-C1 | *Mse*I-24 | 74.22 | 74 | At1g14880 |
| 6 | *Bst*YI-T4 | *Mse*I-31 | 138.8 | 139 | At1g16730 |
| 7 | *Bst*YI-T4 | *Mse*I-32 | 254.44 | 254 | At1g17840 |
| 8 | *Bst*YI-T3 | *Mse*I-2 | 67.5 | 67 | At1g25370 |
| 9 | *Bst*YI-T3 | *Mse*I-21 | 113.9 | 114 | At1g54020 |
| 10 | *Bst*YI-T3 | *Mse*I-32 | 92.2 | 92 | At1g55320 |
| 11 | *Bst*YI-T2 | *Mse*I-23 | 386.35 | 386 | At1g61990 |
| 12 | *Bst*YI-C2 | *Mse*I-12 | 147.4 | 146*(-1.40) | At1g65820 |
| 13 | *Bst*YI-C4 | *Mse*I-12 | 234.3 | 234 | At1g73680 |
| 14 | *Bst*YI-C3 | *Mse*I-2 | 162.55 | 162*(-0.55) | At1g74450 |
| 15 | *Bst*YI-C1 | *Mse*I-2 | 168.8 | 169 | At1g75680 |
| 16 | *Bst*YI-T1 | *MseI-*1 | 399.9 | 400 | At2g06850 |
| 17 | *Bst*YI-T1 | *Mse*I-12 | 83.74 | 84 | At2g16480 |
| 18 | *Bst*YI-C4 | *Mse*I-23 | 160.77 | 161 | At2g26620 |
| 19 | *Bst*YI-C4 | *Mse*I-1 | 313.3 | 313 | At2g31510 |
| 20 | *Bst*YI-T1 | *Mse*I-31 | 169.38 | 169 | At2g32000 |
| 21 | *Bst*YI-T4 | *Mse*I-3 | 154.14 | 154 | At2g36020 |
| 22 | *Bst*YI-T4 | *Mse*I-22 | 162.84 | 163 | At2g40810 |
| 23 | *Bst*YI-T3 | *Mse*I-12 | 110.5 | 111 | At2g45315 |
| 24 | *Bst*YI-C3 | *Mse*I-1 | 110.28 | 110 | At3g09840 |
| 25 | *Bst*YI-T2 | *Mse*I-12 | 106.04 | 106 | At3g17000 |
| 26 | *Bst*YI-T3 | *Mse*I-4 | 325.6 | 326 | At3g21465 |
| 27 | *Bst*YI-T3 | *Mse*I-4 | 209.8 | 210 | At3g22260 |
| 28 | *Bst*YI-C4 | *Mse*I-13 | 105.8 | 106 | At3g24530 |
| 29 | *Bst*YI-T2 | *Mse*I-21 | 202.6 | 203 | At3g25530 |
| 30 | *Bst*YI-T4 | *Mse*I-3 | 240.47 | 240 | At3g29360 |
| 31 | *Bst*YI-T1 | *Mse*I-12 | 169.55 | 170 | At3g47250 |
| 32 | *Bst*YI-C3 | *MseI-*1 | 95.48 | 95 | At3g51280 |
| 33 | *Bst*YI-C4 | *Mse*I-31 | 169.4 | 169 | At3g55250 |
| 34 | *Bst*YI-C4 | *Mse*I-13 | 103.3 | 103 | At3g57510 |
| 35 | *Bst*YI-C4 | *Mse*I-11 | 178.12 | 178 | At3g59380 |
| 36 | *Bst*YI-C1 | *Mse*I-24 | 308.38 | 309*(+0.62) | At4g00180 |
| 37 | *Bst*YI-C3 | *Mse*I-11 | 332.9 | 333 | At4g01000 |
| 38 | *Bst*YI-C1 | *Mse*I-21 | 208.4 | 207*(-1.40) | At4g16830 |
| 39 | *Bst*YI-T3 | *Mse*I-24 | 96.96 | 97 | At4g25315 |
| 40 | *Bst*YI-T4 | *Mse*I-13 | 126.79 | 127 | At4g29450 |
| 41 | *Bst*YI-T4 | *Mse*I-24 | 123.25 | 123 | At4g33450 |
| 42 | *Bst*YI-C2 | *Mse*I-2 | 171.8 | 172 | At4g37530 |
| 43 | *Bst*YI-C1 | *Mse*I-2 | 295.46 | 295 | At5g04895 |
| 44 | *Bst*YI-C1 | *Mse*I-22 | 103.17 | 103 | At5g16620 |
| 45 | *Bst*YI-T4 | *Mse*I-32 | 273.66 | 274 | At5g17080 |
| 46 | *Bst*YI-T1 | *Mse*I-2 | 235.8 | 236 | At5g35730 |
| 47 | *Bst*YI-C1 | *Mse*I-22 | 193.6 | 193*(-0.60) | At5g35737 |
| 48 | *Bst*YI-T4 | *Mse*I-31 | 268.65 | 269 | At5g38320 |
| 49 | *Bst*YI-T4 | *Mse*I-3 | 278.87 | 279 | At5g39510 |
| 50 | *Bst*YI-C2 | *Mse*I-1 | 154.83 | 154*(-0.83) | At5g40240 |
| 51 | *Bst*YI-C4 | *Mse*I-12 | 206.2 | 206 | At5g47800 |
| 52 | *Bst*YI-T3 | *Mse*I-4 | 188.9 | 189 | At5g63330 |
| 53 | *Bst*YI-T1 | *Mse*I-21 | 73 | 73 | At2g39580/At3g05620 |
| 54 | *Bst*YI-T1 | *Mse*I-21 | 242.7 | 243 | At3g24780/At2g45720 |
| 55 | *Bst*YI-T4 | *Mse*I-24 | 107.95 | 108 | At5g10530/At1g36510 |
| 56 | *Bst*YI-T3 | *Mse*I-33 | 210.6 | 211 | At4g00190/At1g33415 |
| 57 | *Bst*YI-T4 | *Mse*I-34 | 97.7 | 98 | At1g04840/At3g56220 |
| 58 | *Bst*YI-T1 | *Mse*I-4 | 168 | 168 | At5g59750/At3g21470 |
| 59 | *Bst*YI-C1 | *Mse*I-24 | 155.55 | 155 | At5g40770/At2g06050 |
| 60 | *Bst*YI-C2 | *Mse*I-1 | 141.48 | 141 | At4g01060/At1g65065 |
| 61 | *Bst*YI-T2 | *Mse*I-14 | 263.6 | 265*(+1.40) | At4g15390/At2g17480 |
| 62 | *Bst*YI-T2 | *Mse*I-21 | 286.2 | 285*(-1.20) | At3g44670/At3g20200 |
| 63 | *Bst*YI-T2 | *Mse*I-23 | 121.8 | 122 | At3g11540/At2g15580 |
| 64 | *Bst*YI-T3 | *Mse*I-12 | 77.25 | 77 | At5g37840/At1g14400 |
| 65 | *Bst*YI-T3 | *Mse*I-34 | 249.26 | 249 | At1g13090/At4g31680 |
| 66 | *Bst*YI-C4 | *Mse*I-2 | 141.25 | 141 | At1g60985/At5g41340 |
| 67 | *Bst*YI-T4 | *Mse*I-2 | 219.8 | 220 | At4g31610/At2g40150 |
| 68 | *Bst*YI-T4 | *Mse*I-2 | 309 | 310 | At1g22610/At5g12370 |
| 69 | *Bst*YI-C4 | *Mse*I-12 | 140.5 | 141 | At4g00270/At4g32850 |
| 70 | *Bst*YI-T4 | *Mse*I-13 | 143.62 | 143 | At5g42090/At1g61490 |
| 71 | *Bst*YI-C4 | *Mse*I-31 | 129.01 | 129 | At1g34210/At2g29240 |
| 72 | *Bst*YI-C4 | *Mse*I-32 | 108.58 | 109 | At2g25490/At4g02550 |
| 73 | *Bst*YI-C4 | *Mse*I-33 | 165.7 | 166 | At2g21800/At4g39210 |
| 74 | *Bst*YI-T1 | *Mse*I-1 | 275 | 275 | No match |
| 75 | *Bst*YI-T1 | *Mse*I-11 | 410 | 410 | No match |
| 76 | *Bst*YI-T1 | *Mse*I-31 | 98.5 | 99 | No match |
| 77 | *Bst*YI-T1 | *Mse*I-2 | 141.67 | 142 | No match |
| 78 | *Bst*YI-T1 | *Mse*I-2 | 335.45 | 335 | No match |
| 79 | *Bst*YI-C2 | *Mse*I-1 | 267.35 | 267 | No match |
| 80 | *Bst*YI-C2 | *Mse*I-14 | 283.18 | 283 | No match |
| 81 | *Bst*YI-T2 | *Mse*I-14 | 314.05 | 314 | No match |
| 82 | *Bst*YI-C2 | *Mse*I-23 | 199.75 | 200 | No match |
| 83 | *Bst*YI-C2 | *Mse*I-23 | 274.81 | 275 | No match |
| 84 | *Bst*YI-T2 | *Mse*I-24 | 131.8 | 132 | No match |
| 85 | *Bst*YI-T2 | *Mse*I-24 | 283.45 | 283 | No match |
| 86 | *Bst*YI-C2 | *Mse*I-24 | 332.32 | 332 | No match |
| 87 | *Bst*YI-C3 | *Mse*I-11 | 187.9 | 188 | No match |
| 88 | *Bst*YI-C3 | *Mse*I-11 | 292.55 | 293 | No match |
| 89 | *Bst*YI-T3 | *Mse*I-12 | 277.29 | 277 | No match |
| 90 | *Bst*YI-T3 | *Mse*I-31 | 205.25 | 205 | No match |
| 91 | *Bst*YI-C3 | *Mse*I-31 | 259.4 | 259 | No match |
| 92 | *Bst*YI-T3 | *Mse*I-32 | 338.22 | 338 | No match |
| 93 | *Bst*YI-T3 | *Mse*I-32 | 398.01 | 398 | No match |
