# Supplementary material for: Identification of imprinted genes subject to parent-of-origin specific expression in Arabidopsis thaliana seeds
Source: BMC Plant Biol. 2011 Aug 12;11:113. doi: 10.1186/1471-2229-11-113 (PMC3174879; doi:10.1186/1471-2229-11-113)
Supplement: Additional file 5 — Table S4 - Lack of detection of known imprinted genes is due to lack of SNPs in restriction sites. MseI cuts in the T/TAA context, Bst YI cuts in the R/GATCY context (source: SALK SNP viewer, TAIR). [file 1471-2229-11-113-S5.DOC]

| **Gene** | **Col x Ler SNPs?** | **Detectable?** | **TDF detected** |
| --- | --- | --- | --- |
|  |  |  |  |
| MEDEA (At1g02580) | None | NA | NA |
| FIS2 (At2g35670) | GGTG[C/G]TTCT  GCGT[T/C]GAGG  GATT[T/G]TGCA  GGTA[G/C]AGTT  TGCA[C/A]AGGT  CCTT[G/A]GCTG | None | NA |
| FWA (At4g25530) | AGTG[C/A]TATC | No | NA |
| MPC (At3g19350) | None | NA | NA |
| PHE1 (At1g65330) | None | NA | NA |
| HDG3 (AT2G32370) | TTTG[T/G]GTGT  GATG[C/T]ATAT  ATTT[G/C]CTTG |  |  |
| HDG8 (At3g03260) | TTTG[A/G]ATAG  TGGT[A/T]ACGC  CACT[T/A]GACC  TGAG[G/A]CAAC  CAAA[G/A]TTAG |  |  |
| HDG9 (At5g17320) | ACTT[G/A]AGCA  CAGC[A/G]CAGA  TCCA[A/T]CGTC | MseI |  |
| *ATMYB3R2* (At4g00540) | GTAT[T/C]TTTG  TTCT[G/C]TGGA  CTTG[C/A]ACAA  TCCT[C/A]TAAG  ATGA[A/C]GAAA  CCCT[T/C]TGAG  ATGT[T/A]ATGC  AGGT[T/C]TAGA  CTGA[A/G]CAAT  GTTT[C/T]CTCT  AGTT[C/T]CTTT  CCTT[A/T]AGCC |  |  |
| *FH5* (At5g54650) | ATTT[T/G]CGAT  TGAG[G/C]ACTT |  |  |
