# Supplementary material for: Identification of imprinted genes subject to parent-of-origin specific expression in Arabidopsis thaliana seeds
Source: BMC Plant Biol. 2011 Aug 12;11:113. doi: 10.1186/1471-2229-11-113 (PMC3174879; doi:10.1186/1471-2229-11-113)
Supplement: Additional file 6 — Table S5 - Relative expression levels of maternally-expressed seed genes in the endosperm and seed coat. Genes detected as maternal by cDNA-AFLP with a log2-ratio of higher than 1 (indicating expression twice as high in endosperm vs. seed coat) are listed. [file 1471-2229-11-113-S6.DOC]

| Gene | log2 of Endosperm / Seed Coat ratio value |
| --- | --- |
| AT3G51280 | 4.646205893 |
| AT1G03070 | 2.491721584 |
| AT2G21130 | 2.467864365 |
| AT5G16620 | 1.961177624 |
| AT5G63330 | 1.829234518 |
| AT5G40390 | 1.812401677 |
| AT4G16830 | 1.428403545 |
| AT3G55250 | 1.330101192 |
| AT1G65820 | 1.260028393 |
