# Supplementary figures and images for: Identification of imprinted genes subject to parent-of-origin specific expression in Arabidopsis thaliana seeds
Source: BMC Plant Biol. 2011 Aug 12;11:113. doi: 10.1186/1471-2229-11-113 (PMC3174879; doi:10.1186/1471-2229-11-113)

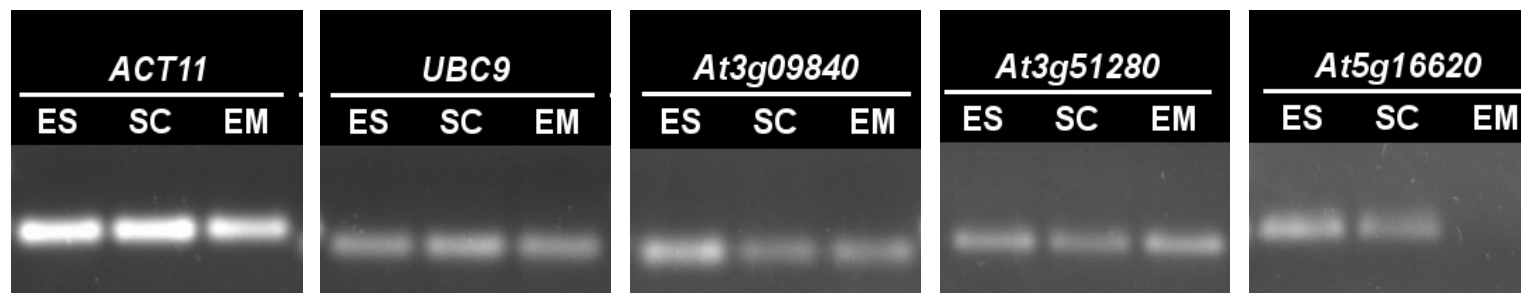

Supplement: Additional file 7 — Figure S2 - Analysis of expression profiles of ATCDC48, PDE120 and MS5-like in LCM tissues of Arabidopsis thaliana seeds (4 dap). Results of RT-PCR performed on cDNA derived from LCM endosperm (ES), seed coat (SC) and embryo (EM) tissues, shown for one representative replicate of two. ACT11 and UBC9 were used as loading controls. [file 1471-2229-11-113-S7.PDF]

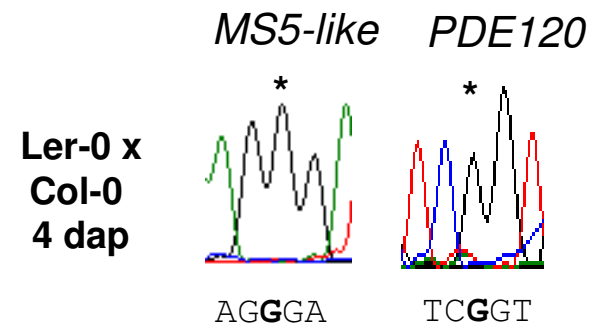

Supplement: Additional file 8 — Figure S3 - Confirmation of maternal expression of cDNA-AFLP candidate genes in crosses to Ler-0. cDNA from 4 dap tissue was amplified and sequenced from F1 Ler-0 × Col-0 hybrid seeds and MS5-like and PDE120 found to be maternally expressed. [file 1471-2229-11-113-S8.PDF]

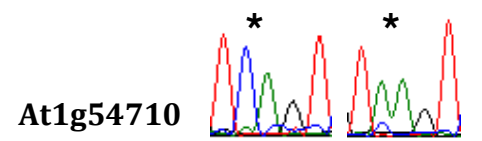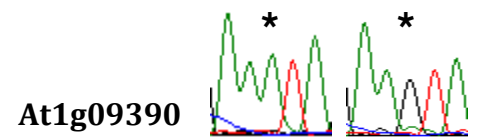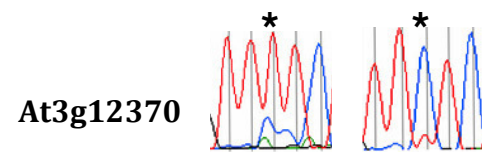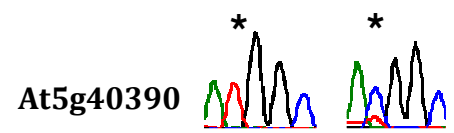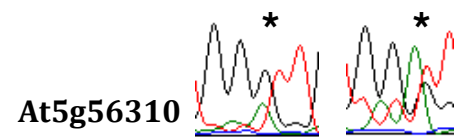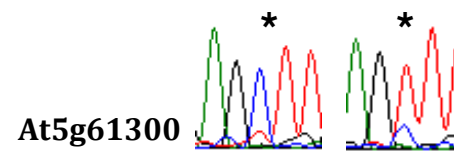

Supplement: Additional file 9 — Figure S4 - Confirmation of six further cDNA-AFLP genes as maternal in seed tissue. cDNA from F1 4 dap seed tissue was amplified and sequenced. In each case, cDNA from Col-0 × C24 is shown on the left, C24 × Col-0 is shown on the right. SNPs are marked with asterisks. [file 1471-2229-11-113-S9.PDF]

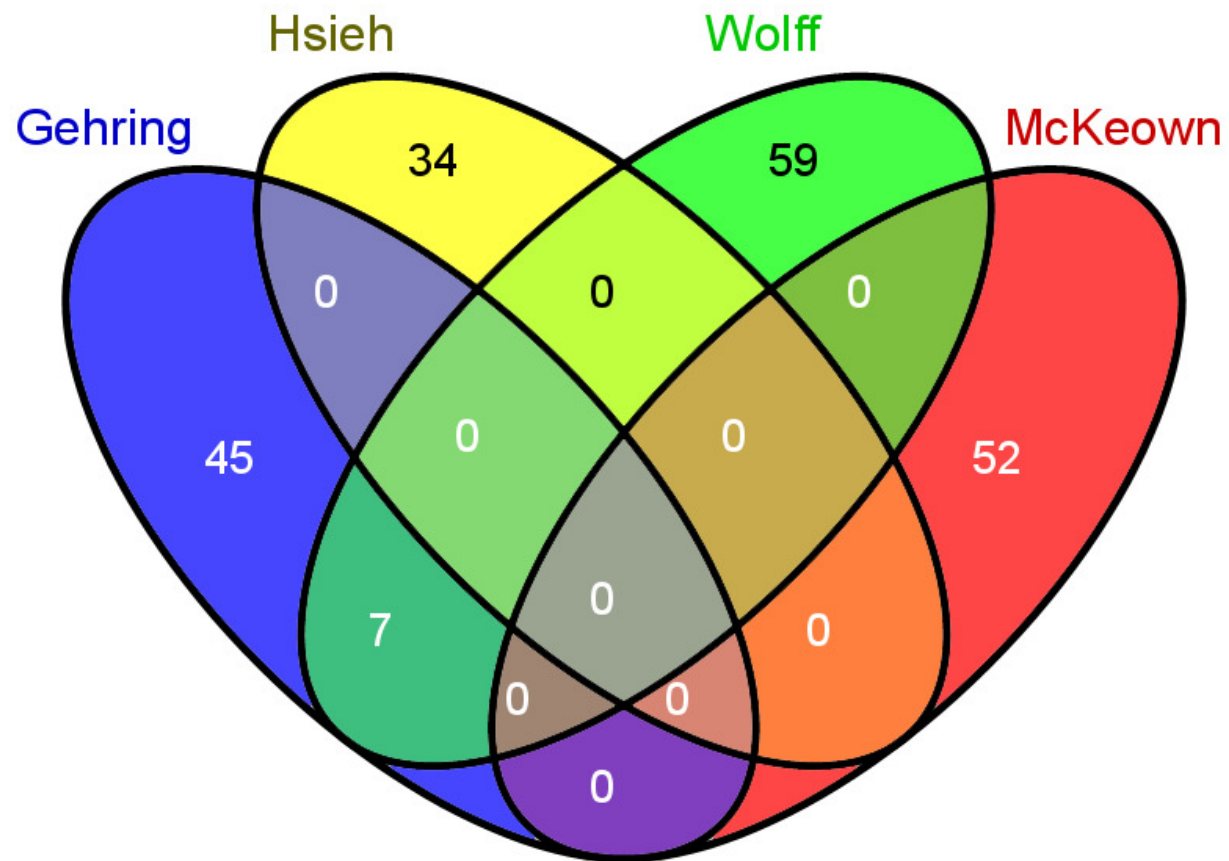

Supplement: Additional file 10 — Figure S5 - Divergent candidate imprinted genes identified by different screens in Arabidopsis. The overlap between maternally-expressed genes identified from this cDNA-AFLP screen performed in 3-5 dap Col-0 × Ler-0 crosses (red); those predicted from analysis of DMRs identified from Col-0 × Ler-0 endosperm (blue, [25]) and those identified by next generation screening approaches ([24] yellow; [23]; green) (see descriptions in Introduction). [file 1471-2229-11-113-S10.PDF]

# AT1G03070

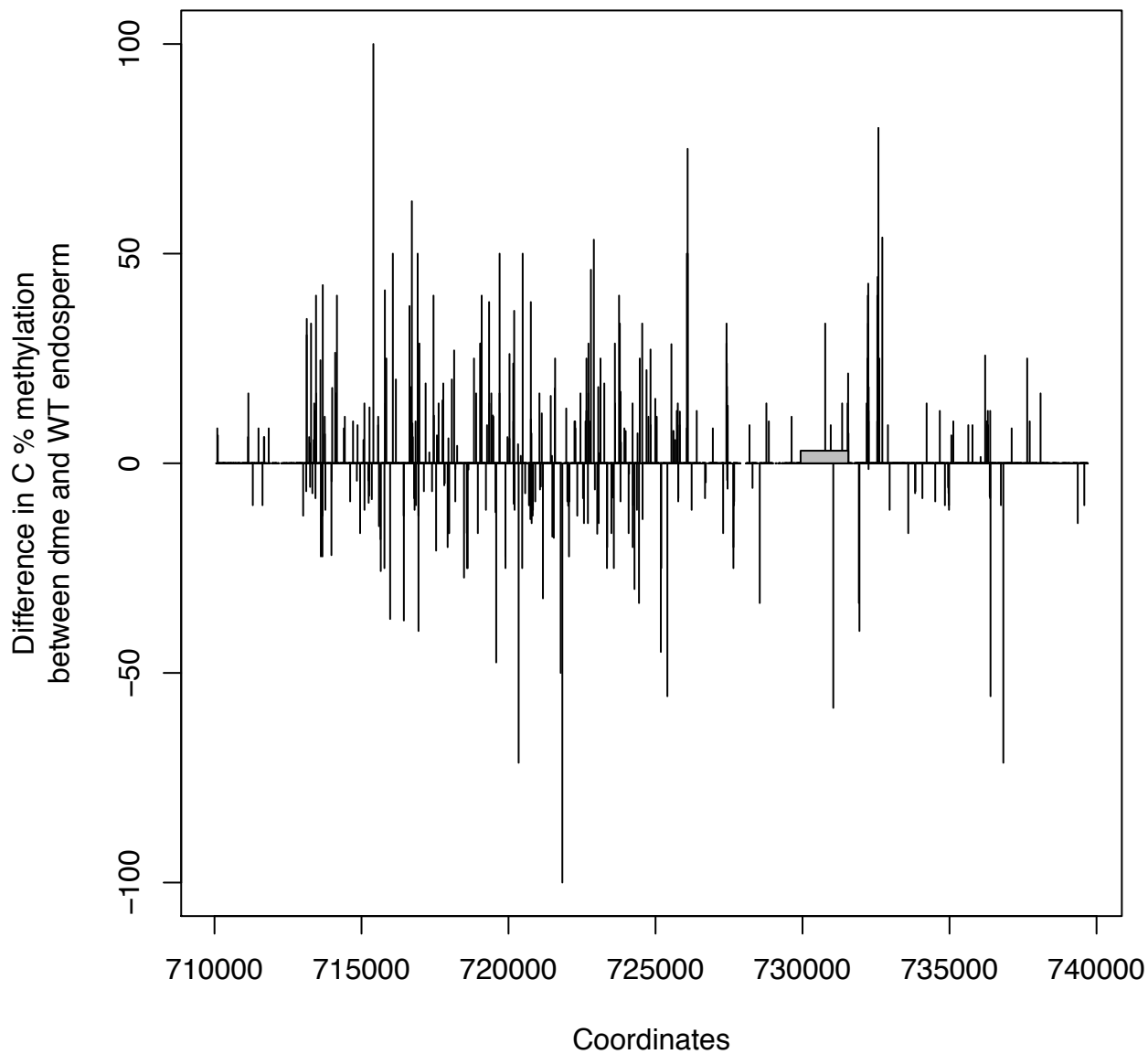

# AT1G25370

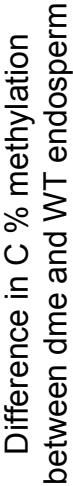

# AT1G61990

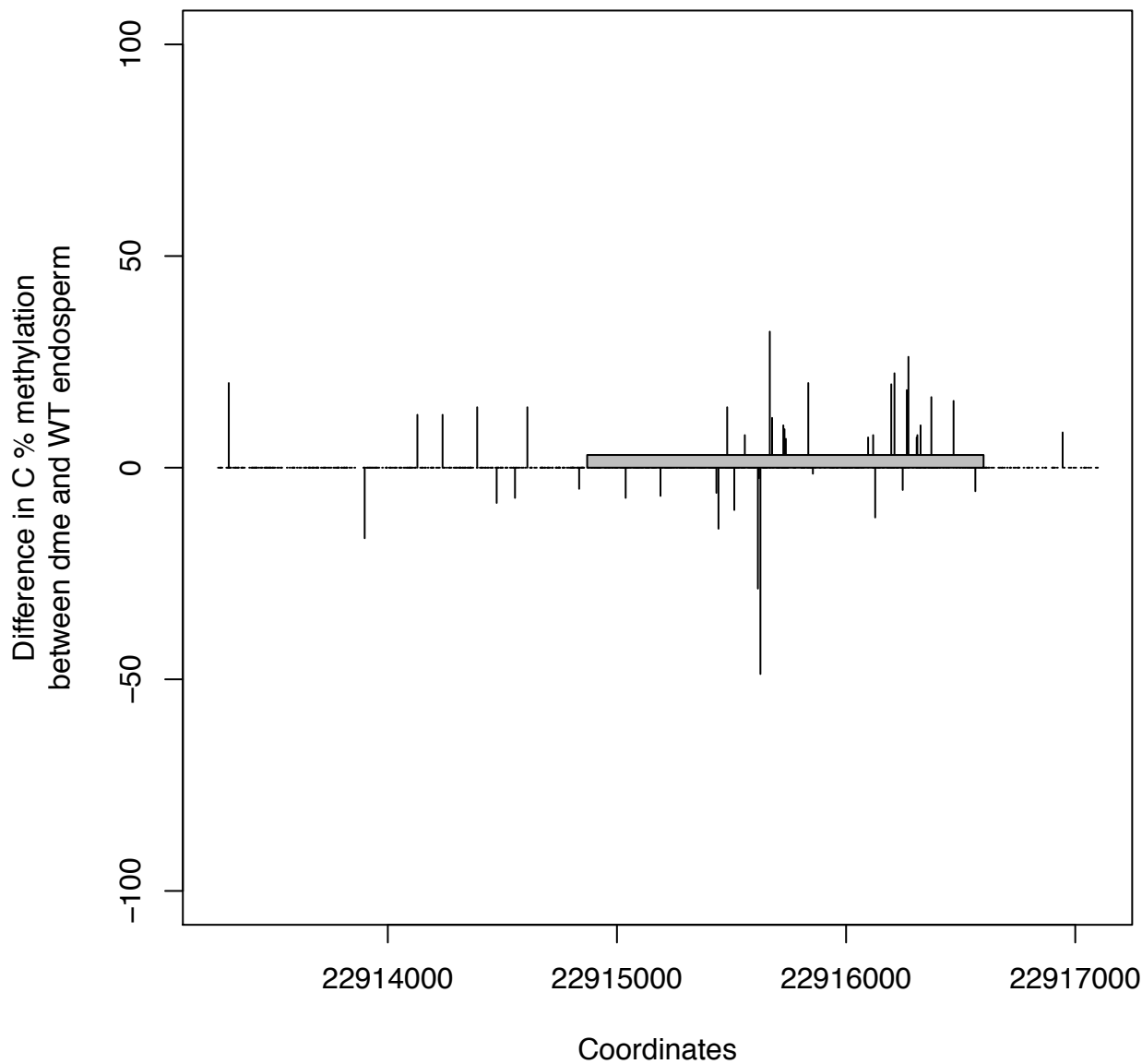

# AT1G65820

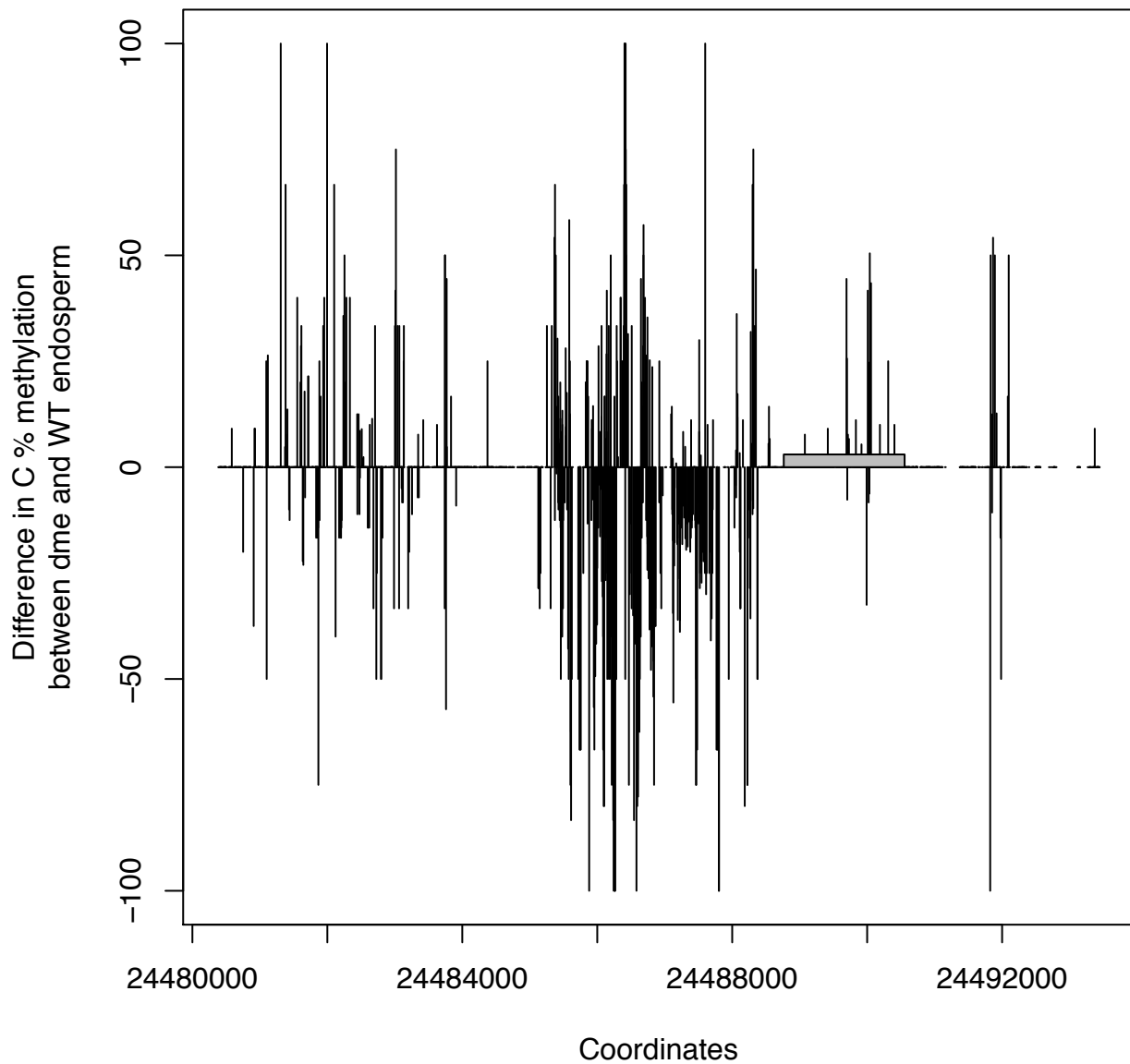

# AT1G73680

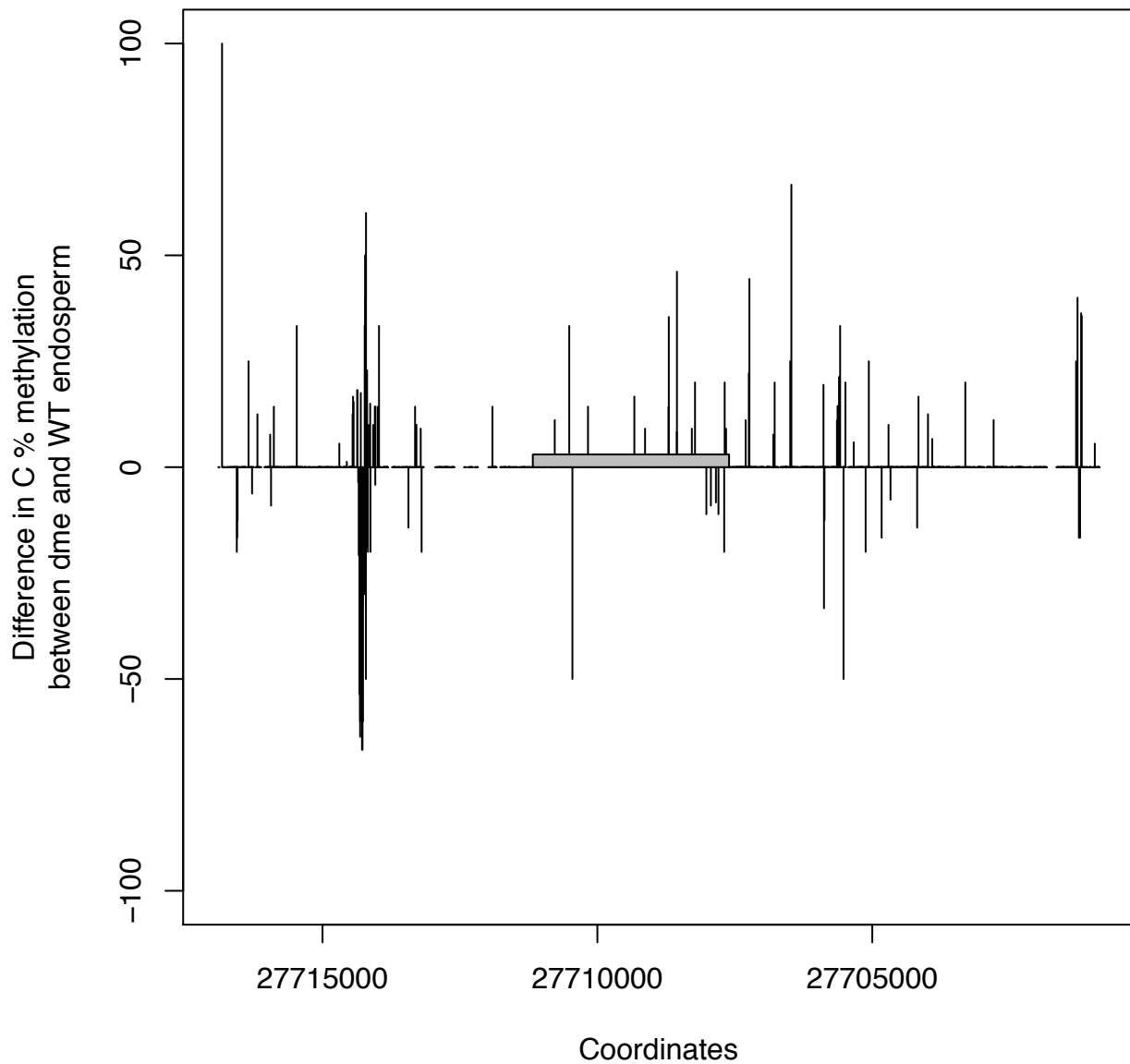

# AT2G16480

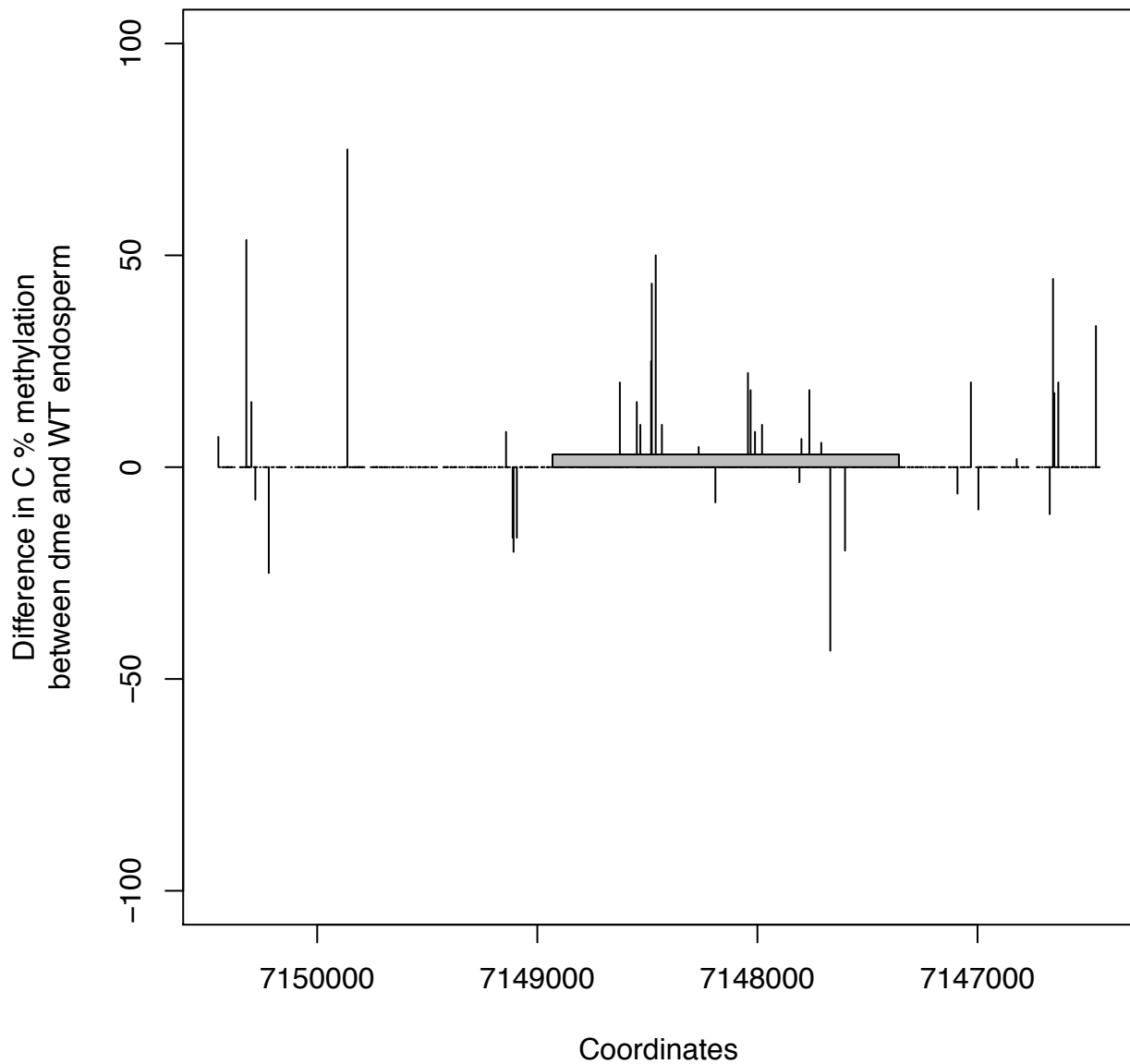

# AT2G31510

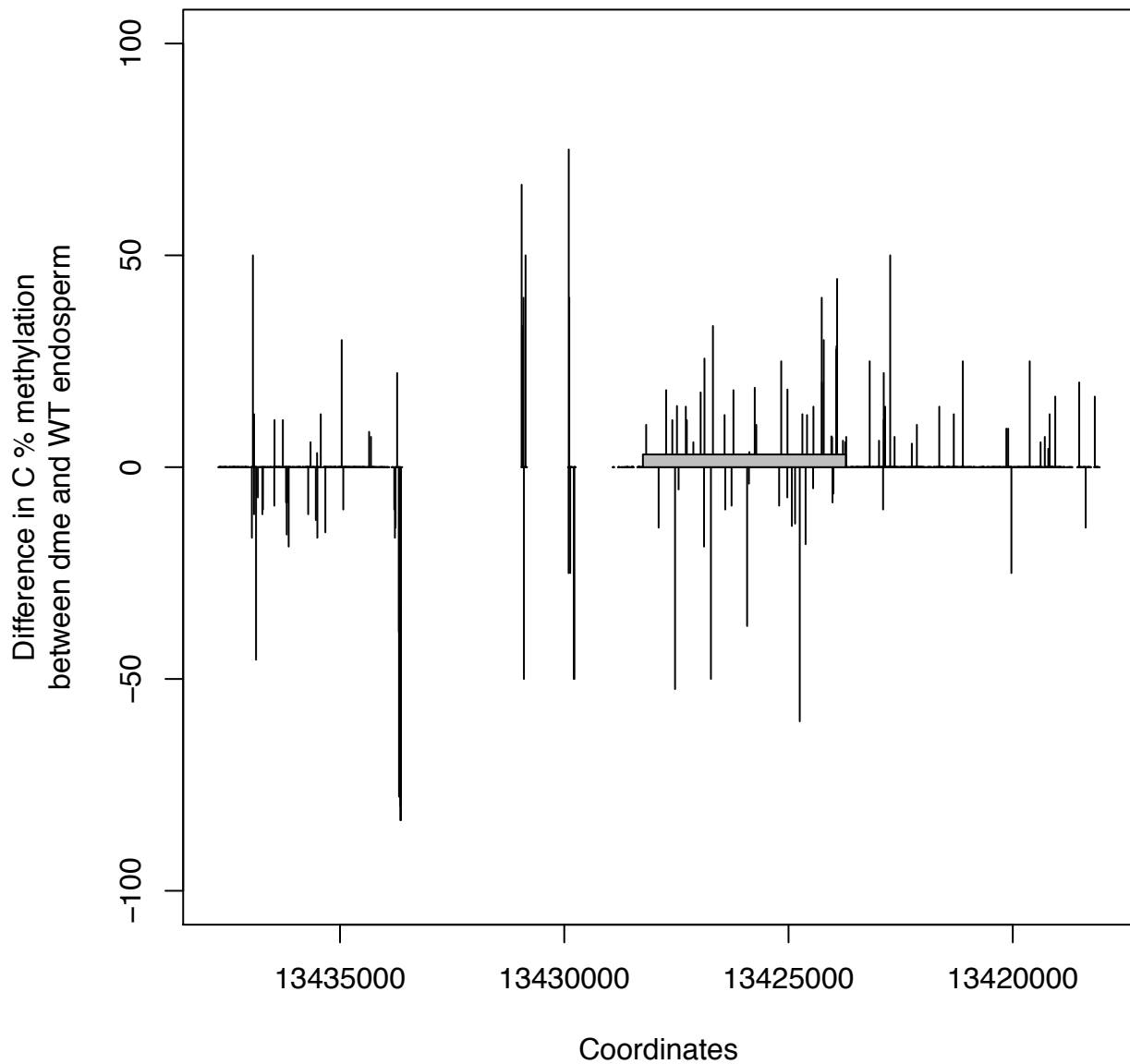

# AT2G32000

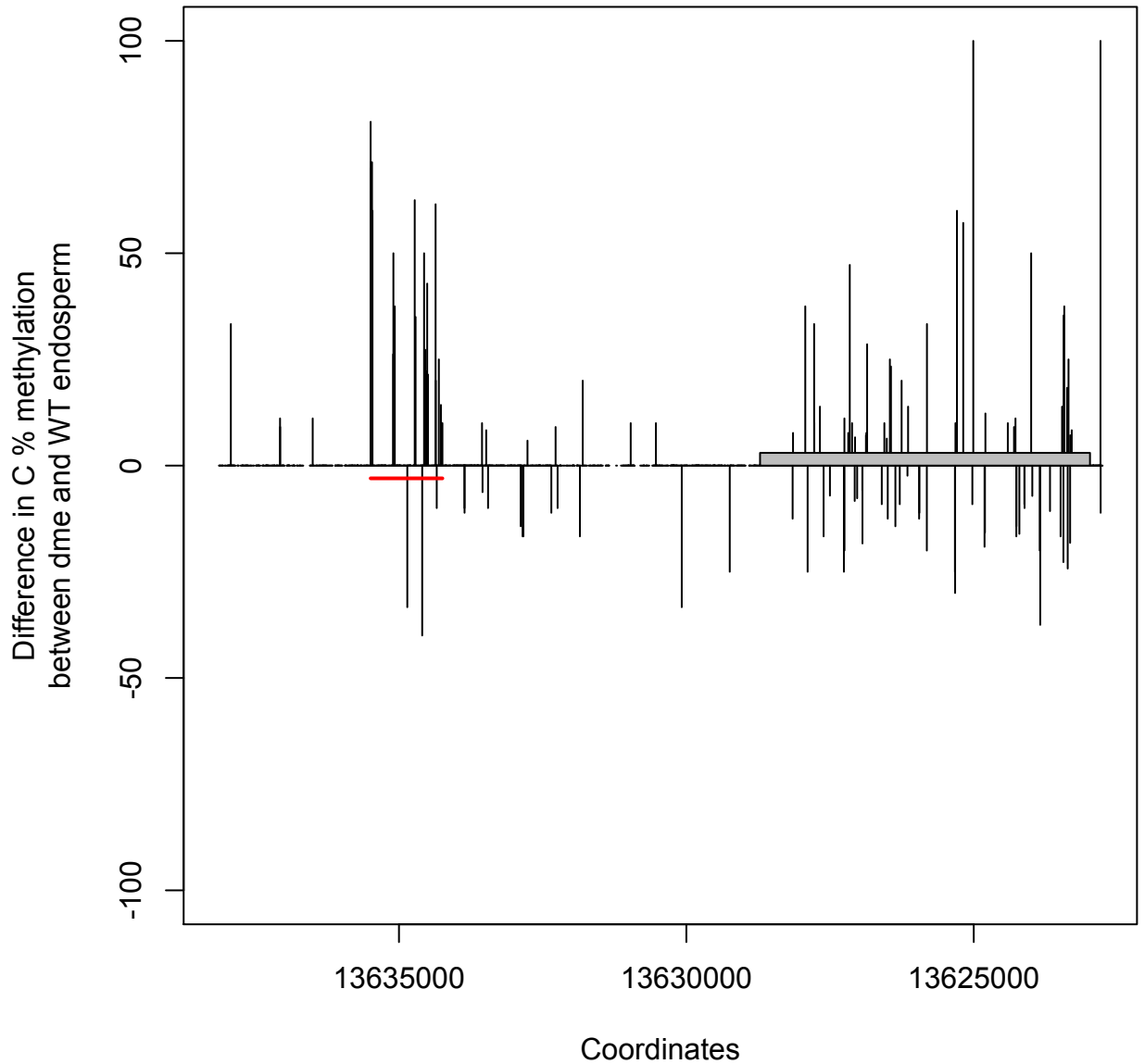

# AT3G17000

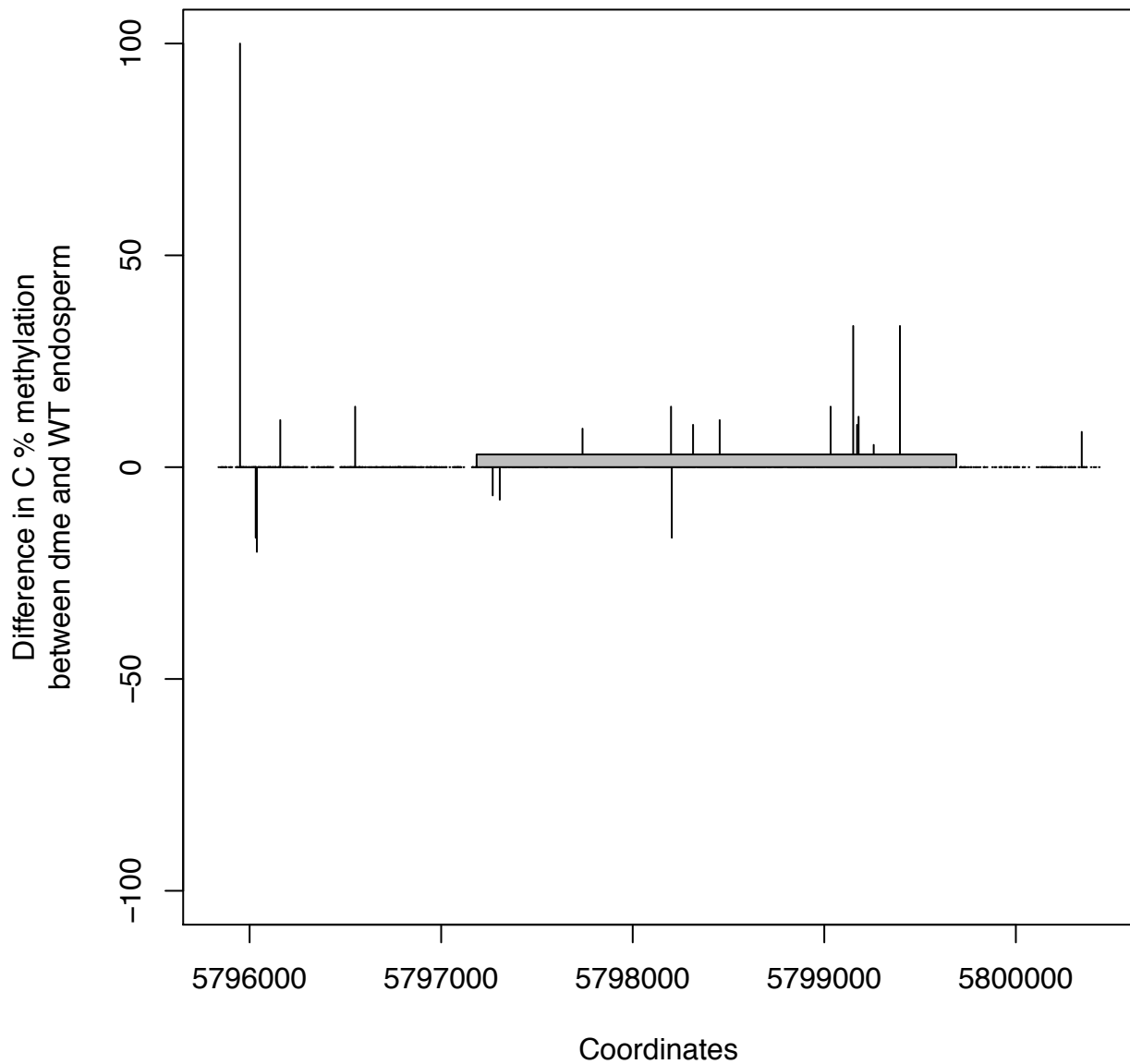

# AT3G24530

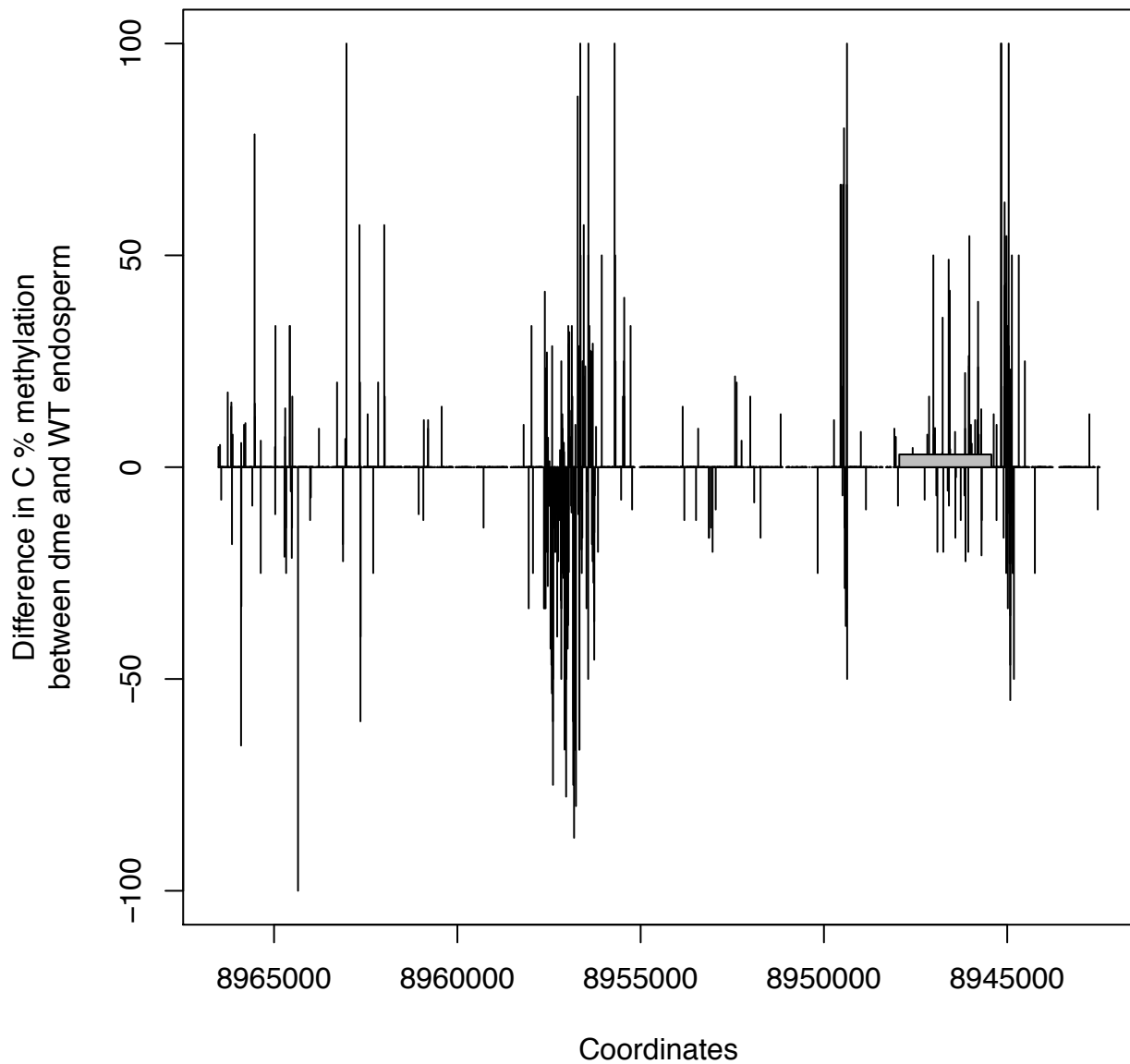

# AT3G55250

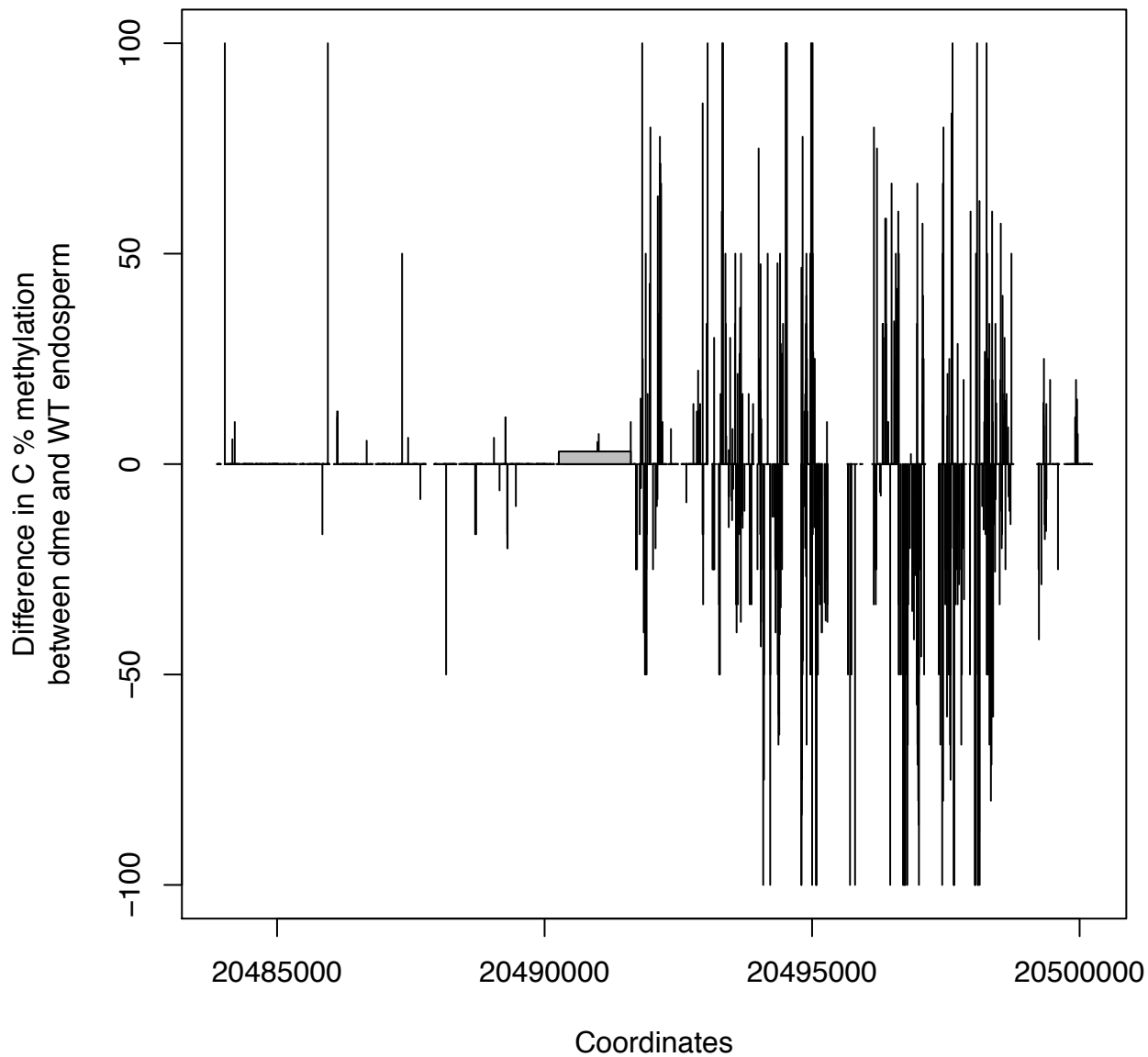

# AT3G59380

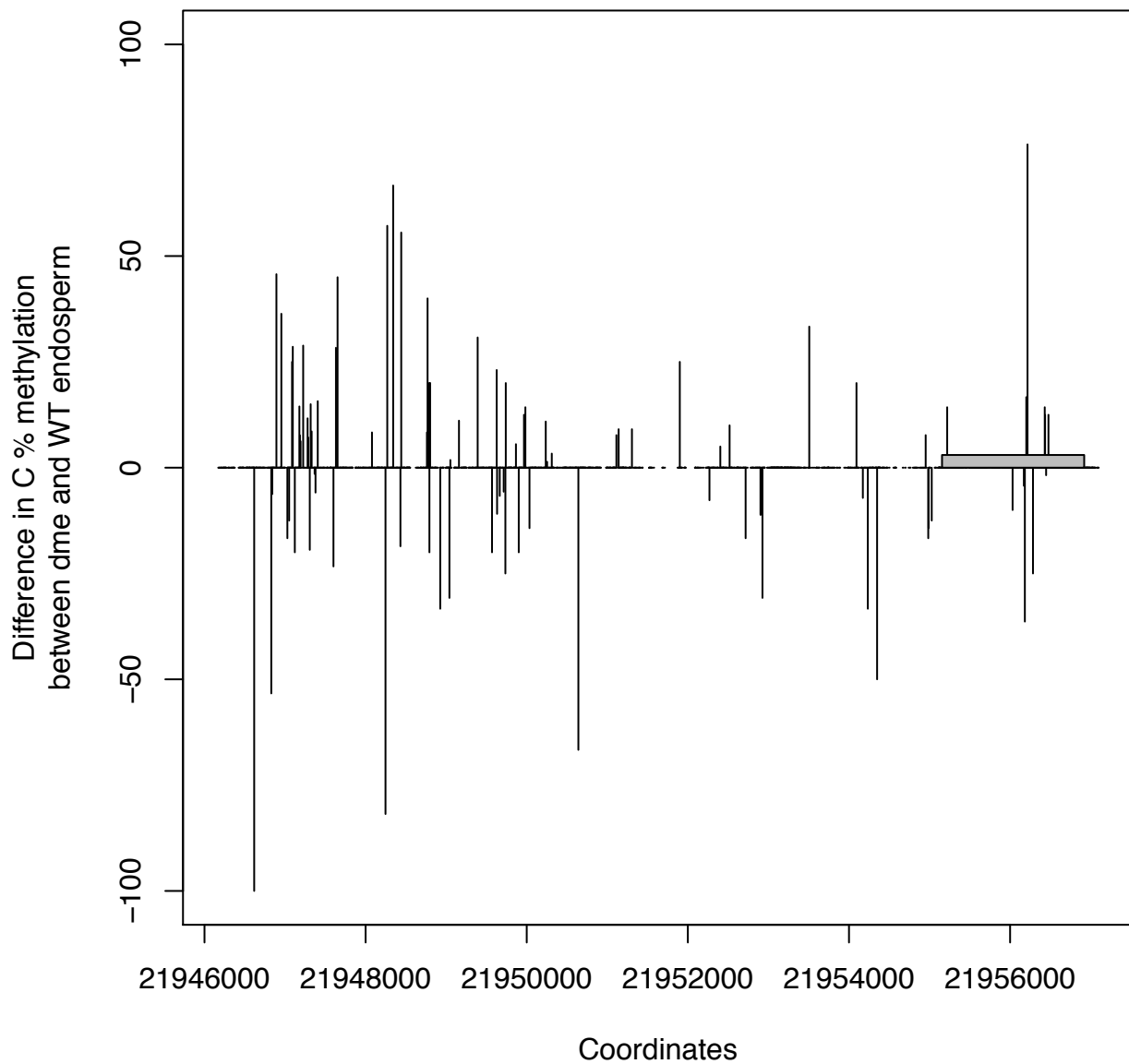

# AT4G16830

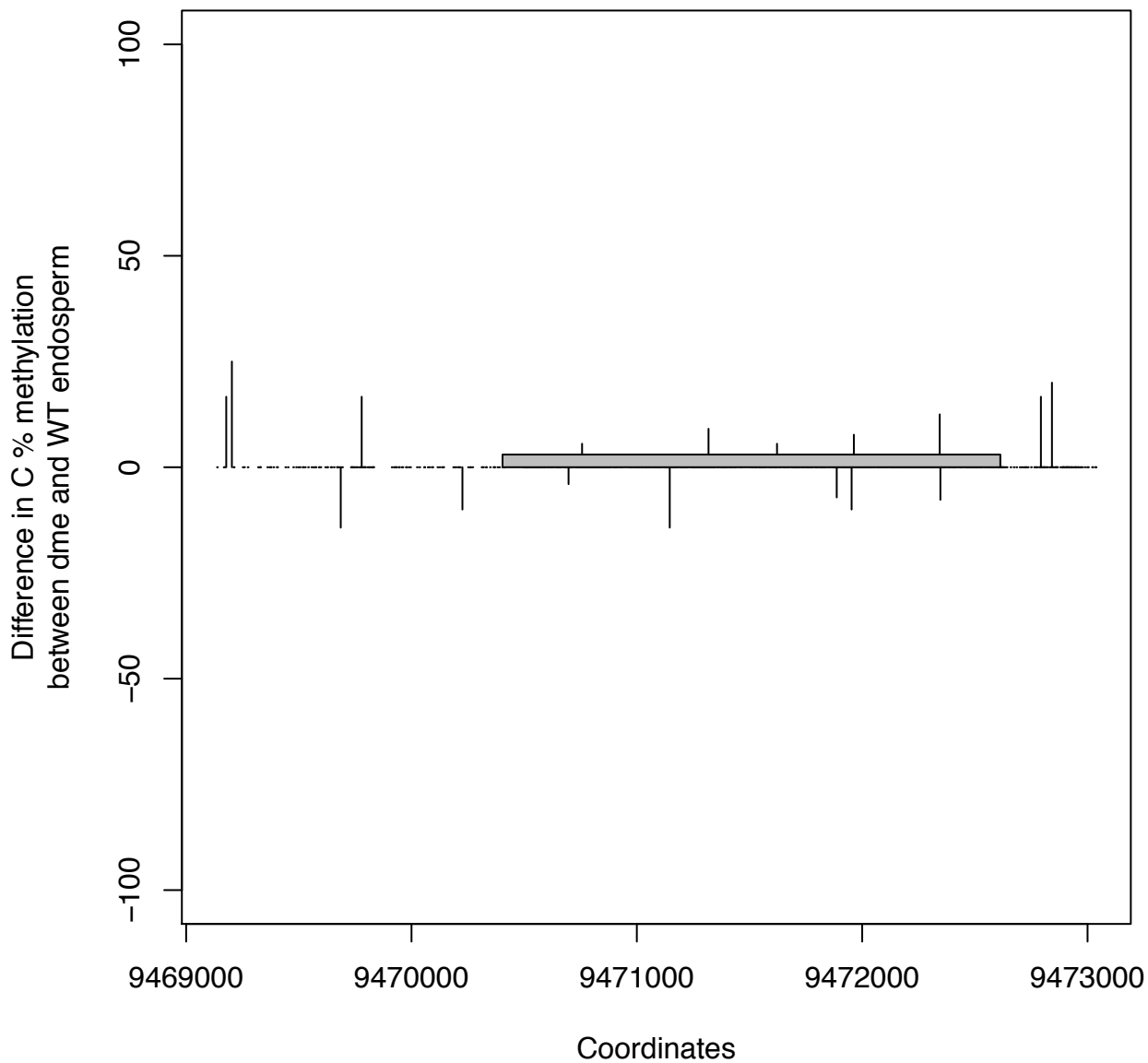

# AT5G39510

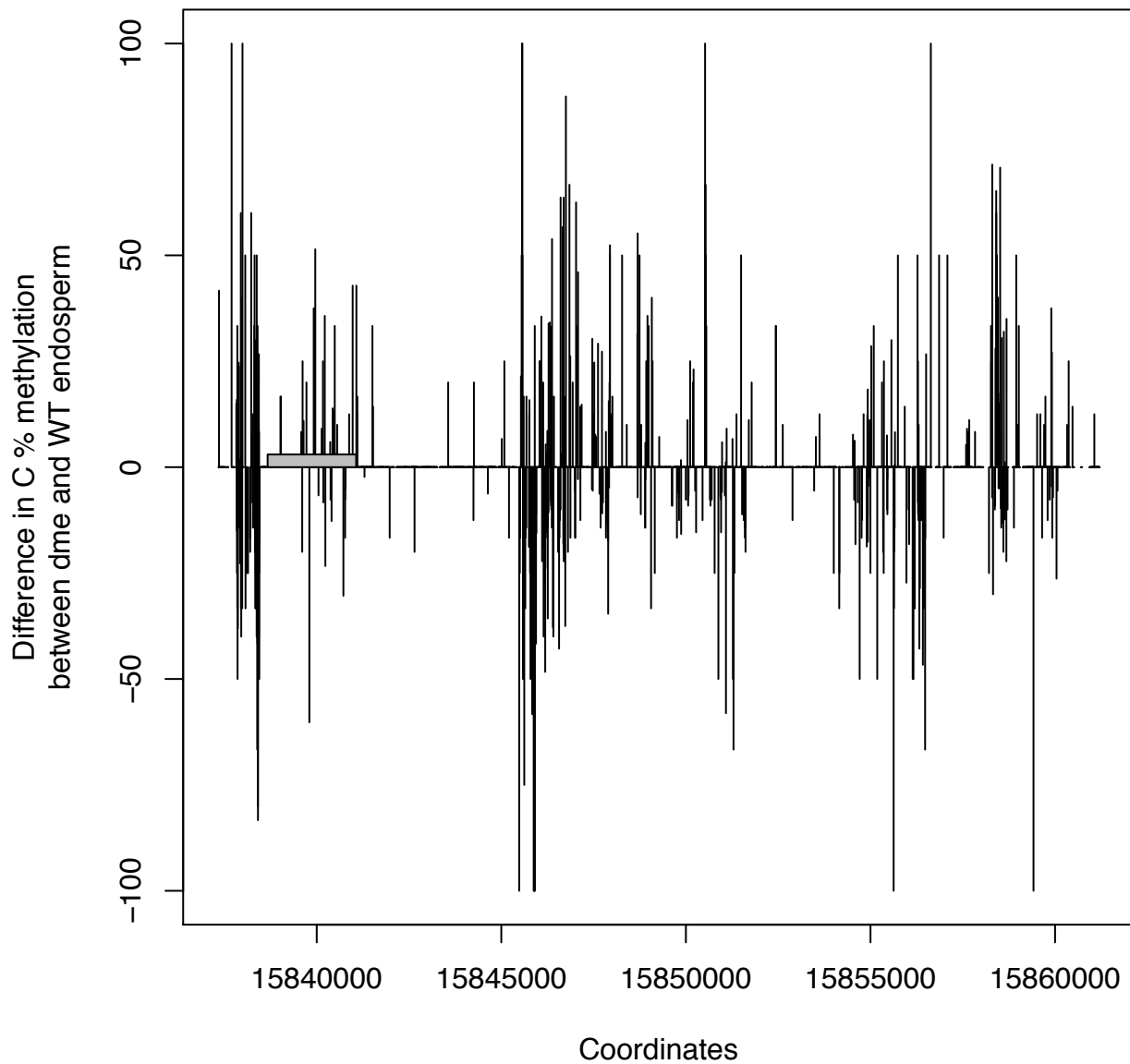

# AT5G63330

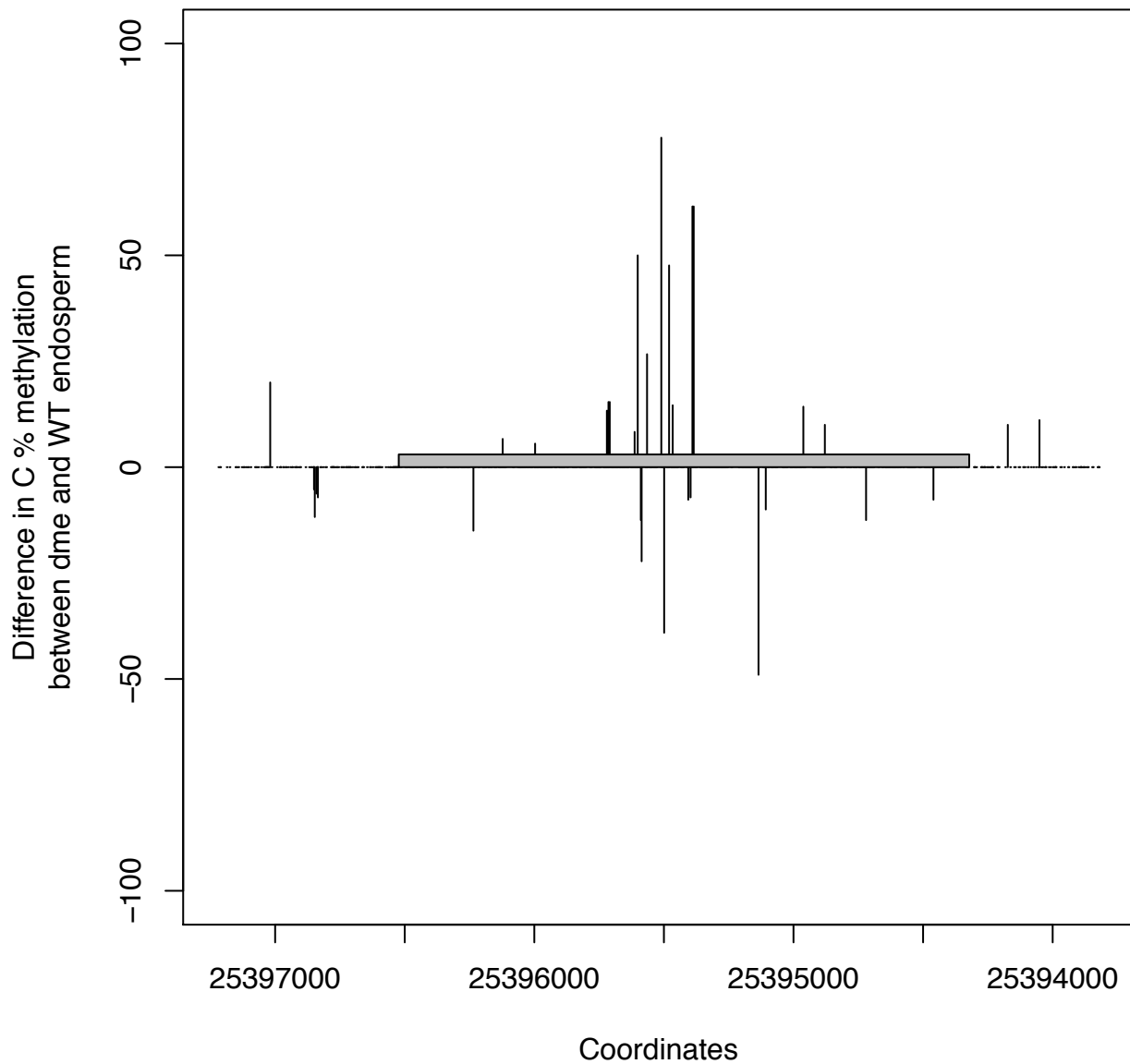

Supplement: Additional file 11 — Figure S5 - Identification of DMRs located in the vicinity of candidate imprinted genes (Table 2) and other maternally inherited genes. Difference in methylation percentage between dme and wild-type endosperm for cytosine bases in the vicinity of three candidate imprinted genes (Table 2). Grey boxes represent the gene body in a 5'-3' orientation, red bars highlight DMRs. [file 1471-2229-11-113-S11.PDF]
